# A Bayesian phylogenetic study of the Dravidian language family

**DOI:** 10.1098/rsos.171504

**Published:** 2018-03-21

**Authors:** Vishnupriya Kolipakam, Fiona M. Jordan, Michael Dunn, Simon J. Greenhill, Remco Bouckaert, Russell D. Gray, Annemarie Verkerk

**Affiliations:** 1 Wildlife Institute of India, Post Box 18, Chandrabani, Dehradun 248001, India; 2 Evolutionary Processes in Language and Culture, Max Planck Institute for Psycholinguistics, Wundtlaan 1, 6525 XD Nijmegen, The Netherlands; 3 Department of Anthropology and Archaeology, University of Bristol, 43 Woodland Road, Bristol BS8 1UU, UK; 4 Department of Linguistic and Cultural Evolution, Max Planck Institute for the Science of Human History, Kahlaische Strasse 10, 07745 Jena, Germany; 5 Department of Linguistics and Philology, Uppsala University, Engelska parken, Thunbergsv. 3 H, 75126 Uppsala, Sweden; 6 ARC Centre of Excellence for the Dynamics of Language, Australian National University, Building 9, H.C. Coombs Bld, Canberra, Australian Capital Territory 2601, Australia; 7 Department of Computer Science, University of Auckland, 303/38 Princes Street, Auckland 1010, New Zealand

**Keywords:** Dravidian, Bayesian phylogenetic inference, BEAST 2, dating, language phylogeny

## Abstract

The Dravidian language family consists of about 80 varieties (Hammarström H. 2016 *Glottolog 2.7*) spoken by 220 million people across southern and central India and surrounding countries (Steever SB. 1998 In *The Dravidian languages* (ed. SB Steever), pp. 1–39: 1). Neither the geographical origin of the Dravidian language homeland nor its exact dispersal through time are known. The history of these languages is crucial for understanding prehistory in Eurasia, because despite their current restricted range, these languages played a significant role in influencing other language groups including Indo-Aryan (Indo-European) and Munda (Austroasiatic) speakers. Here, we report the results of a Bayesian phylogenetic analysis of cognate-coded lexical data, elicited first hand from native speakers, to investigate the subgrouping of the Dravidian language family, and provide dates for the major points of diversification. Our results indicate that the Dravidian language family is approximately 4500 years old, a finding that corresponds well with earlier linguistic and archaeological studies. The main branches of the Dravidian language family (North, Central, South I, South II) are recovered, although the placement of languages within these main branches diverges from previous classifications. We find considerable uncertainty with regard to the relationships between the main branches.

## 1. Introduction

**Figure 1.**
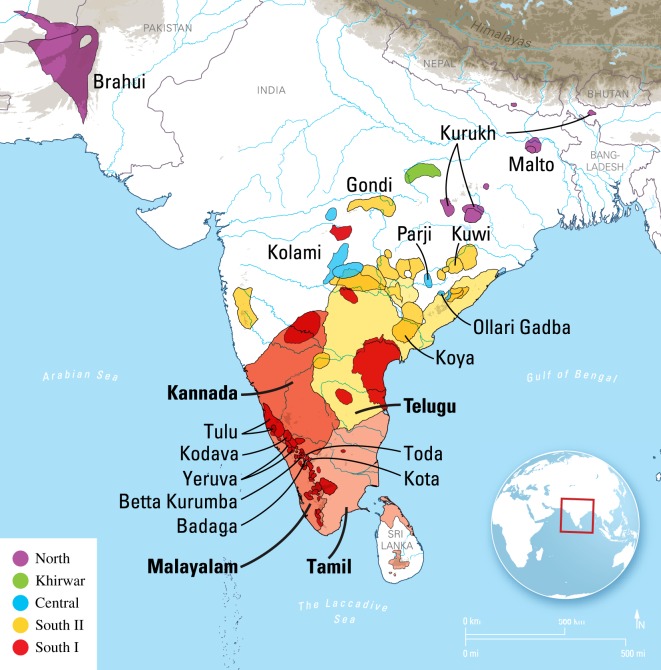
Map of the Dravidian languages in India, Pakistan, Afghanistan and Nepal adapted from *Ethnologue* [2]. Each polygon represents a language variety (language or dialect). Colours correspond to subgroups (see text). The three large South I languages, Kannada, Tamil and Malayalam are light red, while the smaller South I languages are bright red. Languages present in the dataset used in this paper are indicated by name, with languages with long (950 + years) literatures in bold.

The dispersal of human languages and their speakers around the world is often viewed through the lens of large language families. However, the modestly sized Dravidian language family has an important role in resolving questions about the dispersal of human populations throughout Eurasia as well as ancient contact between language families, including Indo-European and Austroasiatic. Dravidian is a language family of around 80 varieties [1] spoken mainly in southern and central India, as well as in a handful of locations in northern India (Kurukh, Malto), Nepal (Kurukh) and Pakistan and Afghanistan (Brahui) (figure 1). These languages range from being spoken by small language communities (Vishavan, 150 speakers), by far larger communities (Kodava, 200 000 speakers), to global languages with literary histories that go back hundreds of years: Malayalam, 33 million speakers; Kannada, 38 million speakers; Tamil, 61 million speakers; and Telugu, 74 million speakers. Dravidian languages have been written for over 2000 years [3], [4, p. 4], [5] have influenced Vedic Sanskrit [6], may have been a part in the formation of all modern Indo-Aryan languages, including even larger languages such as Hindi, Bengali, Punjabi and Marathi [7, pp. 35–42], and are spoken by over 200 million people today [4, p. 4].

Neither the geographical origin of the Dravidian language homeland nor its exact dispersal through time are known. Krishnamurti [7, p. 5] suggests that the Dravidians were ‘natives of the Indian subcontinent who were scattered throughout the country by the time the Aryans entered India around 1500 BCE’. There is linguistic evidence for a much wider dispersal of the Dravidian languages than found today. Most famously, the Indo-European language Marathi, spoken in West-Central India, has substantial input from Dravidian languages indicative of it being spoken in a Dravidian ‘substrate’ environment ([8, pp. 288ff], but this is debated, see [9]). There is clear evidence of Dravidian loanwords into Old-Indo-Aryan (1750–250 BCE) dating to the middle Rigvedic period (*ca* 1200 BCE) in a source area that might have been Sindh, contemporary Southwest Pakistan [6], [8, pp. 69ff, 88]. Southworth [8, p. 64] proposes Sindh, Gujarat and eastern Maharashtra as areas where Dravidian would have been spoken at earlier stages. Krishnamurti [7, pp. 35–42] goes further and suggests a Dravidian substratum affecting Middle Indo-Aryan (Indo-European) and subsequently all modern Indo-Aryan languages. If correct, then this would indicate that these languages arose when Dravidian speakers merged with the Aryan society and learned their language. However, Dravidian speakers were clearly not the first inhabitants of the subcontinent. Southworth [8, pp. 89–90] discusses foreign words and features (e.g. retroflex consonants) that are neither native to Old-Indo-Aryan, nor borrowed from Dravidian or Munda (Afroasiatic). Instead, these features may originate in the South Asian linguistic interaction zone that predates the entry of the Indo-Aryan languages into the subcontinent and that includes languages that have left no known descendants (see additionally on this topic [10, p. 337]; [11, p. 168]; and [12], the latter of which does not refer to the possibility of an extinct substrate).

It has therefore been difficult to assess hypotheses about the Dravidian homeland and dispersal, given the reduced modern-day distribution of the languages from that presumed in pre-Old-Indo-Aryan times. Krishnamurti [7, p. 501] suggests that the Indus civilization, with sites scattered around modern-day Afghanistan, Pakistan and Western India, was Proto-Dravidian, while Parpola [13, p. 174] relates the Indus Script to the Dravidian languages. This is partly based on the notion that the contact with Dravidian in the middle Rigvedic period cannot have been with Proto-North-Dravidian, but may have been with some ancient form of Dravidian. Southworth [8, p. 245] connects Proto-Dravidian with the Southern Neolithic complex, which first appeared in the present Gulbarga, Raichur and Bellary districts of Karnataka, and Kurnool district of Andhra Pradesh around 4500 years ago. However, Southworth [8, p. 249] contends the fit between reconstructed vocabulary items and archaeological remains of crops and cultural items is not perfect, and thus, the link between linguistic and archaeological evidence is not clear [8, p. 256].

The ancient dispersal of the Dravidian speakers is critical to understanding the relationship between the various Dravidian subgroups recognized today. Krishnamurti [7, pp. 18–20] and Southworth [8, p. 50] recognize four main branches: North Dravidian, Central Dravidian, South I Dravidian and South II Dravidian (figure 1). Krishnamurti [7, pp. 492ff] gives a set of 13 sound changes and 27 morphological features (9 nominal, 13 verbal and 5 other) that support these four subgroups, as well as a common stage of South I and South II. South I plus South II Dravidian and Central Dravidian may have shared a common stage, but the evidence for this is minimal [8, pp. 233–236]. Central Dravidian is defined by comparatively few innovations (four, only one of which is a sound change), and Southworth [8, p. 233] emphasizes that even if there is little to no evidence for a common stage of South I + South II Dravidian and Central Dravidian, these two branches must have been in contact at various points in the past to explain several isoglosses cutting across them. Both Southworth [8, p. 235] and Krishnamurti [7, p. 21] (electronic supplementary material, figure S1) opt for a ternary divergence of Proto-Dravidian, into North, Central and South I + South II, which is reflected in the Glottolog reference tree ([1]; see electronic supplementary material, figure S2). However, there is substantial uncertainty in this topology, and Krishnamurti [7, p. 21] is especially uncertain about how the majority of the South I languages relate to each other.

A simplistic view of dispersal and linear genealogical descent of the Dravidian languages is insufficient for understanding their ancient and more recent dispersal. The Dravidian languages have been in contact with each other and their unrelated neighbours for centuries [14]. There exists a major disparity both in prestige and devoted research between smaller, often unwritten languages and the four lingua francas Kannada, Malayalam, Tamil and Telugu, each with literary histories spanning centuries, as well as non-Dravidian state languages including Marathi, Hindi and Desiya (see electronic supplementary material, §6). However, the multi-lingual situation is not simply one where a prestigious state language is learned in school by speakers of smaller languages—Kolami speakers, for instance, may speak Gondi, while most Yeruva speakers are trilingual in both Kodava and Kannada. Even the smaller languages are characterized by their own dialect variation (Gondi [3], Malto [5], Ollari Gadba [15], Koya [16], Tulu [17], Yeruva [18] and Badaga [19]).

Here, we apply Bayesian phylogenetic methods to basic vocabulary data elicited first hand from speakers across the entire language family for this purpose. Basic vocabulary is known to be less likely to be borrowed than other aspects of the lexicon [20]. Rama & Kolachina [21] include an overview of previous quantitative work, which includes various distance-based phylogenetic analysis of morphological and lexical data drawn from existing datasets. Rama & Kolachina [21] itself is a distance-based analysis of lexical data from the *Dravidian Etymological Dictionary*, revised 2nd edition (known as DEDR [22]); lexical data from Krishnamurti [7]; phonological, morphological and syntactic features from Krishnamurti [7]; and Swadesh lists taken from the ASJP project [23]. However, none of these datasets have been gathered with the aim of character-based phylogenetic inference for Dravidian family relationships, as has recently become the standard for similar investigations of other language families, including Austronesian [24], Indo-European [25] and Pama-Nyungan [26]. The current study remedies this gap by combining appropriate first-hand collection of lexical data with sophisticated Bayesian phylogenetic inference methods. The immediate benefit of this approach is that we infer a set of highly supported language family trees, rather than a single best fit, enabling us to capture uncertainty in genealogical patterning.

## 2. Material and methods

### 2.1. Materials

We collected 100 items of basic vocabulary from native speakers of a diverse sample of Dravidian languages. Swadesh's 100-concept elicitation list [27] was used to collect lexical data for 20 languages, listed in table 1. For the purposes of the analysis, we made a point of sampling a sufficient number of varieties to represent all the previously reported subgroups of Dravidian. Data elicitation took place by presenting each informant (all native speakers of the relevant language, except for Malto and Betta Kurumba) with the Swadesh list on paper in the commonly used literary language for that particular language. Care was taken to provide the right context of each concept [30]. The informants were asked to say the word for each comparative concept in their native language. Responses were recorded and written down if this was possible. For some unwritten languages, informants did not want to write down responses in non-native scripts. Recordings and written transcripts were transcribed to the International Phonetic Alphabet (IPA).

**Table 1.** Data sources of languages included in the study.

language	ISO code	Glottocode	classification according to Krishnamurti [7]	source form	native speaker	primary source	number of missing Swadesh items
Brahui	brh	brah1256	North	written (Arabic, Latin Scripts), audio	Abdul Raziq, Balochistan	Kolipakam [28]	0
Malto	mjt	saur1249	North	written (Arabic Script), audio	—	C. Puttaswamy (personal communication; 2010)	5
Kurukh	kru	kuru1302	North	written (Arabic Script), audio	AK Baxla, Jharkhand	Kolipakam [28]	0
Ollari Gadba	gdb	pott1240	Central	audio	Rajesh (elicited by MK Mishra, Orissa)	Kolipakam [28]	41
Parji	pci	duru1236	Central	audio	Mohan (elicited by MK Mishra, Orissa)	Kolipakam [28]	36
Kolami	kfb	nort2699	Central	audio	Karan, Orissa	Kolipakam [28]	4
Kuwi	kxv	kuvi1243	South-II	audio	Anup (elicited by MK Mishra, Chhattisgarh)	Kolipakam [28]	44
Gondi	gno	nort2702	South-II	written (Latin Script), audio	Pawan, Madhya Pradesh	Kolipakam [28]	0
Koya	kff	koya1251	South-II	written (Telugu Script), audio	Peter Daniels, Andhra Pradesh, Khammam district	Kolipakam [29]	0
Telugu	tel	telu1262	South-II	written (Telugu Script), audio	V. Kolipakam, Andhra Pradesh	Kolipakam [29]	0
Tamil	tam	tami1289	South-I	written (Tamil, Telugu Scripts), audio	Venkatachalam Chokkalingam, Tamil Nadu	Kolipakam [29]	0
Malayalam	mal	mala1464	South-I	written (Malayalam, Telugu Scripts), audio	Anil Nair, Kerala	Kolipakam [29]	0
Kannada	kan	nucl1305	South -I	written (Kannada, Telugu Scripts), audio	Ponnappa, Karnataka	Kolipakam [29]	0
Kodava	kfa	koda1255	South -I	written (Kannada, Telugu Scripts), audio	Bopanna, Karnataka	Kolipakam [29]	0
Tulu	tcy	tulu1258	South -I	written (Kannada, Telugu Scripts), audio	Sunanda, Karnataka	Kolipakam [29]	2
Yeruva	yea	ravu1237	South-I	audio	Chubakki, Karnataka	Kolipakam [29]	1
Toda	tcx	toda1252	South-I	audio	Kishore, Tamil Nadu	Kolipakam [29]	0
Kota	kfe	kota1263	South-I	written (Tamil, Telugu Scripts), audio	Mohanraj, Tamil Nadu	Kolipakam [28]	9
Badaga	bfq	bada1257	South-I	written (Tamil, Telugu Scripts), audio	Yellapa, Tamil Nadu	Kolipakam [29]	5
Betta Kurumba	xub	bett1235	South-I	IPA transcription	—	G. Coehlo (personal communication; 2010)	0

Critically, this methodology is an improvement on previous studies of the Dravidian family, including Rama & Kolachina [21] and Borin *et al*. [14], who use pre-existing comparative datasets derived from dictionaries. Collecting data first hand with the contexts of the required concept provided as described above addresses three issues. First, dictionaries will often provide multiple translations for a word, which may artificially inflate the number of cognate sets with deep histories [31]. Second, dictionaries of lesser-known languages may have many gaps. These factors can create artificial clusters in the analysis: languages with more speakers or long literary histories will have better descriptions in dictionaries, which may cause ‘large’ languages to cluster together in phylogenetic analysis. At the other extreme, languages with a lot of missing data may also be clustered together. A third difference is that we are modelling the history of synonymous cognate sets, i.e. groups of related words for the same concept, whereas what is taken from etymological dictionaries are proper cognates sets, which may have undergone change in meaning.

The primary reason for collecting first-hand data was therefore methodological. In addition, existing lexical comparative datasets for Dravidian are limited. Grierson [32] features 19 Dravidian languages; Beine [33] is a survey of Gondi dialects in 46 locations (see [34] for a computational analysis). The *Dravidian Etymological Dictionary*, revised 2nd edition [22] features information on 29 languages, but is heavily skewed towards written languages, and especially towards the four largest Dravidian languages Kannada, Malayalam, Tamil and Telugu. The lexicons of Dravidian languages are generally under-described, with only 15 out of 80 varieties listed on Glottolog [1] to have a dictionary, plus 12 more with word lists that are not Grierson [32] or Beine [33]. See also [35, pp. 106–107] on the status of description of the Dravidian family.

We add to table 1 the number of missing comparative concepts for each language. For Kuwi, Ollari Gadba and Parji, elicitation of comparative concepts was problematic because in several cases, we could not be sure that the speaker had understood the correct Swadesh context due to a missing common language. Additionally, in cases where multi-lingual speakers did not know the word in their native language, but used a word from another language they spoke, responses were not recorded (see electronic supplementary material, §6 on multi-lingualism).

Cognate coding of the responses was performed using the *Dravidian Etymological Dictionary*, revised 2nd edition [22]. For words where cognacy information was not available, judgements on cognacy were based on sound changes from Proto-Dravidian to the contemporary languages as listed in Burrow & Emeneau [22]. Errors by the informants or transcription errors were excluded from the analysis. Loan words listed as borrowings in Burrow & Emeneau [22] as well as clear loans from Indo-European languages were excluded from the analysis. Multi-state cognate-coded words for each comparative concept were transformed into binary-coded cognate sets with each site representing whether a cognate in that particular cognate set is present (1), absent (0) or missing (?) in each language. The dataset contains 778 sites of which 91% are complete. It has been published as Kolipakam *et al*. [36] and is available online.

### 2.2. Methodology

#### 2.2.1. Robustness of cognate coding

To provide a further check on cognate coding, we used new methods designed to aid robustness of linguistic inferences. Sound sequences in the original data were adjusted with help of orthography profiles [37], ‘segments’ package) that provide a simple means to segment and correct phonetic transcriptions. To increase the future comparability of the data, all concepts were linked to the Concepticon [38]. An automatic cognate detection analysis of the data was performed with LingPy (v. 2.5.1, [39]) using the LexStat-Infomap algorithm [40]. The standard threshold of 0.55 [40, p. 8] yielded precision values of 0.90 and an overall *F*-score of 0.84 (using B-cubed evaluation scores), giving 90% agreement between our cognate coding and the automatic cognate detection algorithm. The code and files for the automatic cognate detection analysis have been made available as electronic supplementary material.

#### 2.2.2. Model of evolution

We used BEAST 2 (v. 2.4.3, [41]) for all phylogenetic analyses. We tested three different models of cognate gain and loss. The first cognate model was a simple binary continuous time Markov chain (CTMC) model that is essentially the generalized time reversible model [42] for binary data [43], and allows cognates to be gained and lost at the same rate. The second cognate model was a binary covarion [44] which allows each cognate set to remain relatively stable over time but occasionally switch into a faster rate category to undergo bursts of change on different branches. The third cognate model was a stochastic Dollo [45,46] that allows cognates to appear once on a tree but get lost multiple times (i.e. Dollo's Law).

To account for rate variation across branches, we fitted two different clock models. The first clock model is a strict clock that assumes no rate variation over time. The second clock model was a relaxed clock [47] where branch rates were sampled from a lognormal distribution with parameters estimated from the data. We performed ascertainment correction to compensate for the fact that only sites are included in the cognate alignment that have at least one 1 in them, taking missing data in account [31]. Further, we tested for the case where individual concept classes have their own relative mutation rate estimated versus keeping them fixed at 1, as proposed by Chang *et al*. [31]. With a relative rate of 1, all concepts are assumed to evolve at the same overall rate. This has been the common practice in previous language phylogeny work. With ‘word rates’, each site (=cognate set) in a concept class has its rate shared with other sites in the same concept class, but each concept class has its own rate. This adds more complexity to the model, but models the rate variation between comparative concepts. For the CTMC model, γ rate heterogeneity with four rate categories was investigated as well [48].

The tree prior consists of uniform priors over subgroups (see below) and the Yule prior, which is governed by the birth rate. The prior on the birth rate is uniform (0,1), so the birth rate is bounded. The combination of these priors guarantees that the resulting prior on the tree is a proper prior: it cannot collapse to zero height due to lower bounds, and cannot escape to infinity due to upper bounds. This makes it possible to perform stepping stone analyses [49] reliably with the combined tree prior.

We ran each analysis for 100 million generations, sampling trees every 5000 generations to remove autocorrelation, resulting in a sample of 20 000 trees. A burn-in of 10% of the iterations was removed leaving a posterior sample of 18 000 trees. For estimating marginal likelihoods, we used a stepping stone analysis [49] as implemented in BEAST 2 with 64 steps and 1 million samples per step. Autocorrelation and convergence checks were carried out using Tracer v. 1.6 [50]. Maximum clade credibility (MCC) trees were generated using TreeAnnotator [51].

#### 2.2.3. Subgrouping and dating

To estimate the age of the Dravidian language family, we incorporated historical information as priors on language divergence times. These priors allow the clock parameter to make inferences on chronological time using rates of cognate gain and loss. Calibrations were based on information on phonological and morphological innovations (Brahui, North, South I, South II) and the first attestations of the written Dravidian languages in cave inscriptions and literary works (Kannada, Malayalam, Tamil, Telugu, Tulu). We specified these dates as amount of years in the past, taking 2000 as the date for the present. Additionally, we included several priors on subgrouping. We placed constraints on the North, South I and South II groups to be monophyletic as these have well-documented shared phonological and morphological innovations. On South I, we included a calibration so that this group could not be younger than 2250 years, because Tamil was recorded first in 254 BCE [4, p. 6]. Constrained subgroup priors and calibrations were implemented as probabilistic priors on the relevant nodes as indicated in table 2; further details are given in the electronic supplementary material, §1.

**Table 2.** Calibration points.

	languages involved	prior
1	all 20 languages	lower bound of 0 yr, upper bound of 10 000 yr
2	North: Brahui, Kurukh, Malto	monophyletic constraint only; no age associated
3	South I: Badga, Betta Kurumba, Kannada, Kodava, Kota, Malayalam, Tamil, Tulu, Toda, Yeruva	lower bound of 2250 yr
4	South II: Gondi, Koya, Kuwi, Telugu	monophyletic constraint only; no age associated
5	the divergence of Brahui	upper bound of 2250 yr
6	the divergence of Malayalam	normal distribution centred on 1000 yr with standard deviation of 50, truncated to 800–1200 yr
7	the divergence of Telugu	lower bound of 1400 yr
8	the divergence of Kannada–Tulu	lower bound of 1300 yr, not monophyletic

In order to further assess the certainty associated with the higher-order subgrouping, we investigated two further sets of calibrations. Regime (1) had no monophyletic constraints, because we removed calibration points 2, 3 and 4, in order to assess the tree topology obtained without subgrouping constraints. Regime (2) had an extra monophyletic calibration on South I and South II, to test Krishnamurti's idea [7, pp. 495–497] that South I and South II languages form a clade. We used the highest ranking model of evolution for these two analyses, as below.

## 3. Results

### 3.1. NeighborNet network

**Figure 2.**
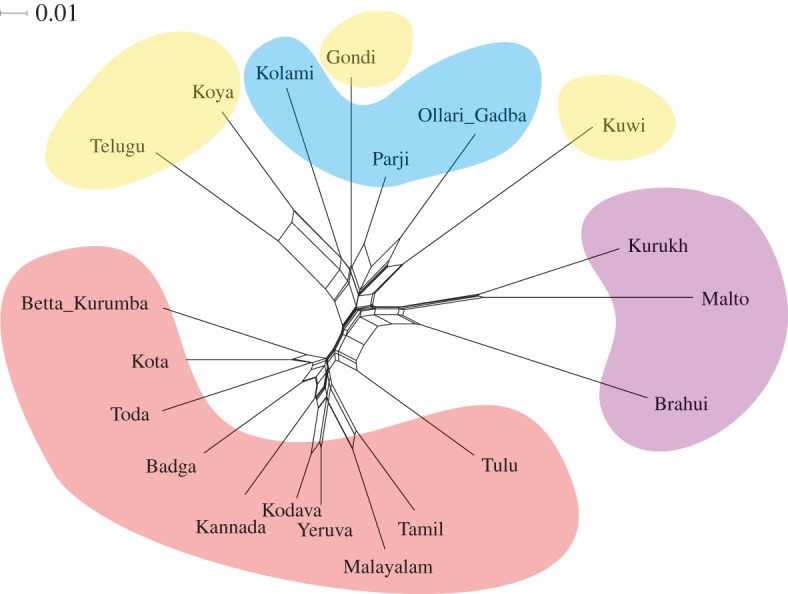
A NeighborNet visualization of lexical differences. The NeighborNet network identifies three groups, going in clockwise direction starting from Telugu in the upper left corner: South II + Central (Telugu, Koya, Kolami, Gondi, Parji, Ollari Gadba and Kuwi), North (Kurukh, Malto and Brahui) and South I (Tulu, Tamil, Malayalam, Yeruva, Kodava, Kannada, Badga, Toda, Kota and Betta Kurumba). Colour-coding gives subgroup affiliation: red, South I; blue, Central; purple, North; yellow, South II.

To visualize potential conflicting signal in our lexical dataset, we first constructed a NeighborNet network (figure 2) using SplitsTree v. 4.14.4 [52]. The network was calculated from the cognate-coded data using Hamming distances between pairs of languages. The size of the boxes at the core of the network is a measure of conflicting signal (such as borrowing) between the languages. Two other measures of conflicting signal calculated by SplitsTree, δ scores and *Q*-residuals, are included in the electronic supplementary material, §3, and discussed below.

While the NeighborNet network clearly distinguishes three groups, reticulate signal is evident for Tulu, which is located on the edge of the South I subgroup and shows some affinity with Brahui, and Telugu and Koya. These visual patterns are corroborated by two statistics calculated on the network, the δ score [53] and the *Q*-residual [54]. The mean δ score is 0.30, Telugu and Tulu have higher scores, suggesting greater contribution to reticulate patterns (Telugu: 0.38; Tulu: 0.35). Telugu also has a high *Q*-residual score, 0.0132, almost twice as high as the mean of 0.0069. The mean δ score and *Q*-residual score are not different to what has been reported for other families: Polynesian [54], described as having substantial conflicting signal, δ score 0.41, *Q*-residual 0.02; Cariban [55] δ score 0.36; Austronesian [54] δ score 0.33, *Q*-residual 0.002; Indo-European [54] δ score 0.22, *Q*-residual 0.002. We believe at least a part of the conflicting signal can be explained by the multi-lingual situations in which speakers of nearly all Dravidian languages find themselves. As an illustration, we present other languages commonly spoken in each language community, along with other sociolinguistic information, in electronic supplementary material, §6. The consequences of these sociolinguistic patterns are further elaborated upon in Discussion.

### 3.2. Model of evolution

**Figure 3.**
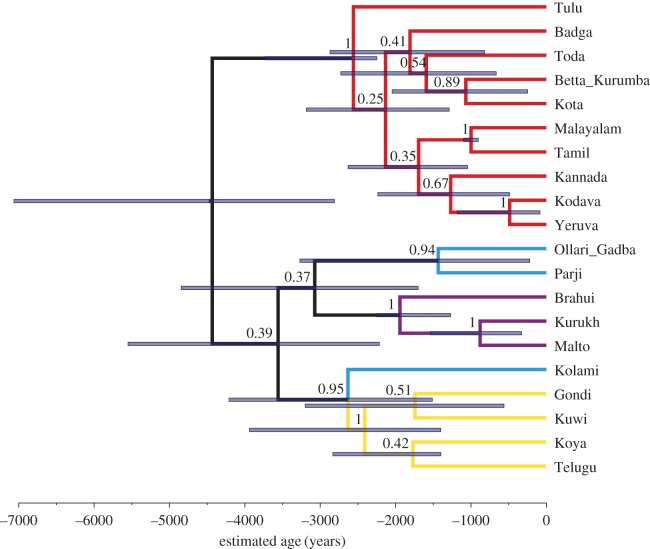
MCC tree summary of the posterior probability distribution of the tree sample generated by the analysis with the relaxed covarion model with relative mutation rates estimated. Node bars give the 95% highest posterior density (HPD) limits of the node heights. Numbers over branches give the posterior probability of the node to the right (range 0–1). Colour coding of the branches gives subgroup affiliation: red, South I; blue, Central; purple, North; yellow, South II.

To identify the best-fitting model of cognate evolution and clock model, we calculated marginal likelihoods using path sampling [49,56], as implemented in BEAST 2, and Bayes factors [57], given in table 3. The best performing model was the covarion model with relaxed clock and individual mutation rates estimated (marginal L = −4128), which showed positive support over the next best model, the CTMC analysis with relaxed clock and estimated mutation rates (marginal L = −4131). The MCC tree of the best-fitting model, including posterior support values for the nodes and a scale giving time in years before present, is shown in figure 3. A DensiTree [58] is given in the electronic supplementary material, §4, to illustrate the variation in the posterior sample of trees. The BEAST 2 xml file for the best-fitting model, as well as the resulting set of phylogenetic trees and their MCC tree, have been made available as electronic supplementary material.

**Table 3.** Model comparison results. Models of cognate gain and loss included are (i) CTMC; (ii) CTMC plus γ rate heterogeneity with four rate categories (CTMC + γ); (iii) binary covarion (Covarion); (iv) stochastic Dollo (Dollo). Models of tree rate variation are (i) strict clock (strict); (ii) relaxed clock with rates sampled from lognormal distribution (relaxed). Models of concept rate variation are (i) relative mutation rate estimated for individual concepts (word rates); (ii) no relative mutation rate estimated (overall rate).

analysis	model	clock	mutation rates	marginal log-likelihood	difference	BayesFactor (2 × ln(*K*))
cov-est-relax	Covarion	relaxed	word rates	−4128	—	—
ctmc-est-relax	CTMC	relaxed	word rates	−4131	−3	6
ctmc4 g-est-relax	CTMC + γ	relaxed	word rates	−4136	−4	8
cov-fixed-relax	Covarion	relaxed	overall rate	−4147	−11	22
ctmc4 g-fixed-relax	CTMC + γ	relaxed	overall rate	−4153	−6	12
cov-est-strict	Covarion	strict	word rates	−4182	−29	58
ctmc-est-strict	CTMC	strict	word rates	−4185	−3	6
ctmc4 g-est-strict	CTMC + γ	strict	word rates	−4191	−6	12
ctmc-fixed-relax	CTMC	relaxed	overall rate	−4234	−43	86
sdollo-est-relax	Dollo	relaxed	word rates	−4540	−306	612

### 3.3. Subgrouping

**Figure 4.**
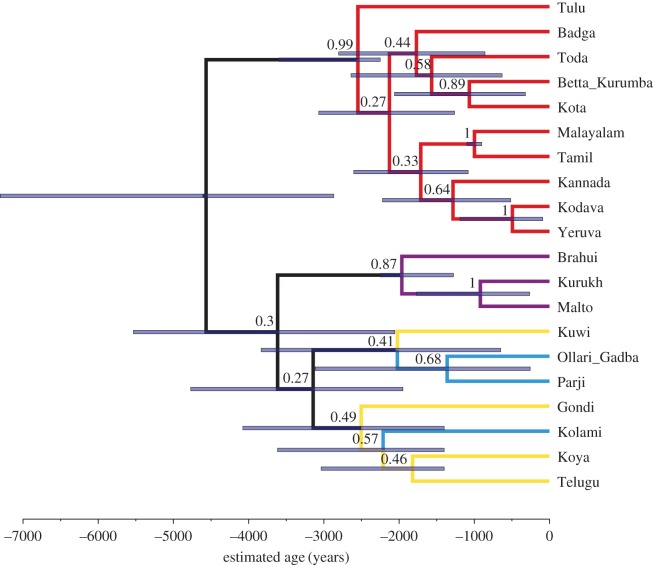
Analysis without monophyletic constraints on the North, South I and South II subgroup. MCC tree summary of the posterior probability distribution of the tree sample, relaxed covarion model with relative mutation rates estimated. Node bars give the 95% highest posterior density (HPD) limits of the node heights. Numbers over branches give the posterior probability of the node to the right (range 0–1). Colour coding of the branches gives subgroup affiliation: red, South I; blue, Central; purple, North; yellow, South II.

**Figure 5.**
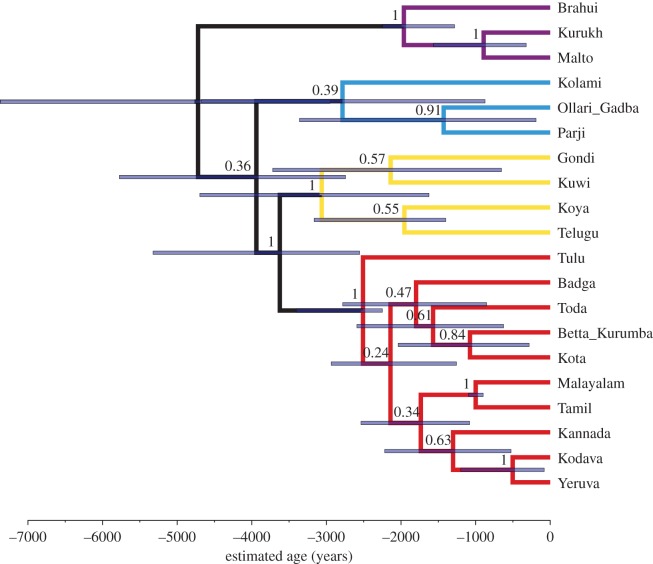
Analysis with additional monophyletic constraints on South I + South II. MCC tree summary of the posterior probability distribution of the tree sample, relaxed covarion model with relative mutation rates estimated. Node bars give the 95% HPD limits of the node heights. Numbers over branches give the posterior probability of the node to the right (range 0–1). Colour coding of the branches gives subgroup affiliation: red, South I; blue, Central; purple, North; yellow, South II.

In the MCC tree of the highest ranking model tree sample (figure 3; hereafter ‘best-supported tree’), we find four main groups, all of which, except Central, were constrained in the priors: South I (Badga, Betta Kurumba, Kota, Toda, Kannada, Kodava, Yeruva, Malayalam, Tamil, Tulu), South II (Gondi, Kuwi, Telugu, Koya, with the Central language Kolami as outgroup), Central (Ollari Gadba, Parji) and North (Brahui, Kurukh, Malto). To discuss this finding, we present the most commonly used reference tree, Krishnamurti's [7, p. 21] family tree, in the electronic supplementary material, figure S1. Following, MCC trees of the two additional analyses ran using the highest ranking model above, the relaxed covarion model with relative mutation rates estimated, with either no monophyletic groups (figure 4) or an extra South I + South II monophyletic group (figure 5). We also include the expert-judgement classification presented in Glottolog [1] in the electronic supplementary material, figure S2, which is derived from Krishnamurti [7, p. 21] and other information. The Glottolog tree corresponds exactly to Krishnamurti's [7] classification, except for the placement of Betta Kurumba, which is closer to Kannada in Glottolog [1].

The structure of the South I group matches previous linguistic hypotheses fairly well. The first match is the close affinity of Tamil and Malayalam as sisters in both trees. The position of Tulu in the best-supported tree matches Krishnamurti [7, p. 21] as it is the first language to split off from the South I group, but it is uncertain (see the DensiTree in the electronic supplementary material, figure S4, see also [59,60]). The hierarchical grouping of Badga, Toda, Kota and Betta Kurumba is present in both trees; one difference is that the close relationship between Badga and Kannada present in Krishnamurti's tree is not supported by ours. The best-supported tree seems to return geographical proximity for South I languages. Out of the smaller South I languages, Kodava and Yeruva are spoken in Kannada territory, so these are likely to have borrowed from the more widely spoken language Kannada. Yeruva and Kodava are neighbouring languages. The other group is formed by Badga, Betta Kurumba, Kota and Toda, which are spoken in very close proximity in the borderlands of Malayalam, Kannada and Tamil.

In the South II group, Koya is sister to Telugu in the best-supported tree, although Hammarström *et al*. [1] place Koya and Gondi together. We find that the Central language Kolami tends to be clustered with the South II group, leaving Ollari Gadba and Parji as the remaining two Central languages: these form a clade sister to the North subgroup. Within the North subgroup, the best-supported tree matches well with Krishnamurti [7, p. 21]: Brahui splits off first, while Malto and Kurukh are more closely related.

Most notably, the higher subgrouping presented in figure 3 diverges from Krishnamurti's [7, p. 21] subgrouping. There, South I and South II are grouped together, so the main three groups splitting off from Proto-Dravidian are North, Central and South I + South II. In our best-supported tree, the main split is between South I and the three other groups. However, note that the support for this grouping is low, with low posterior support for the node connecting South II, Central and North, and the node connecting Central and North. The posterior support for South I and South II in the best-supported tree is low (found in only 42 trees out of 18 000, proportion 0.0023).

We analysed two further calibration regimes that were (i) less and (ii) more constrained. For these, we used the highest ranking model of cognate evolution (relaxed covarion model with relative mutation rates estimated), with either no monophyletic groups (MCC tree presented in figure 4) or an extra South I + South II monophyletic group (figure 5). Regime (1) contained no monophyletic groupings, and only calibration points 1, 5, 6, 7 and 8 from table 2. Under these assumptions, we recover the main split between South I and the other three groups that is found in the more constrained analyses (figures 3 and 5). Support for the higher-order branches in the North + Central + South II group remains low. With the monophyletic constraint on South II released, Kuwi positions as sister to the two Central languages, Ollari Gadba and Parji. Kolami is included in the South II group in this less constrained analysis.

Under regime (2), with monophyletic priors kept in and a ninth calibration constraining South I and South II as monophyletic added (figure 5), we instead recover the more ‘traditional’ topology as hypothesized in Krishnamurti [7, p. 493] and Fuller [61, p. 200] (as based on [62, p. 267]). The North group splits off first, and South I + South II diverges last.

### 3.4. Dating

**Figure 6.**
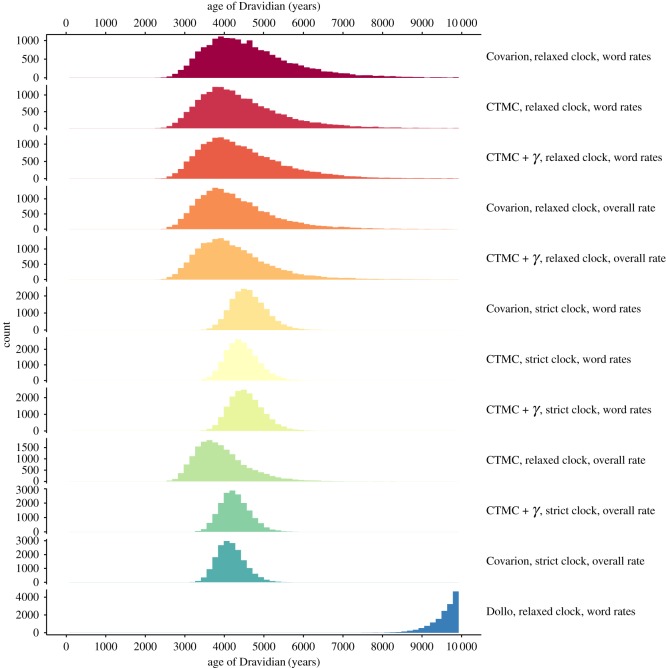
Frequency plots of the age of the Dravidian language family (tree height) for all models, with age in years from the present. Analyses are ordered by marginal log-likelihood, table 3, and electronic supplementary material, table S2. Models of cognate gain and loss included are (i) CTMC; (ii) CTMC plus γ rate heterogeneity with four rate categories (CTMC + γ); (iii) binary covarion (covarion); (iv) stochastic Dollo (Dollo). Models of tree rate variation are (i) strict clock; (ii) relaxed clock with rates sampled from lognormal distribution. Models of concept rate variation are (i) relative mutation rate estimated for individual concepts (word rates); (ii) no relative mutation rate estimated (overall rate).

Root dating of the Dravidian language family using the calibration points captured in table 2 varies only slightly between models, as shown in figure 6. The only model to diverge from a general 95% highest posterior density (HPD) interval on the root of the tree between approximately 3000 and 6500 years ago is the stochastic Dollo model, which performs much worse than all other models (see marginal log-likelihood in table 3). We note that the stochastic Dollo model makes the unrealistic assumption that there is no semantic change—reflexes of real cognate sets may drift in and out of the Swadesh list as their meaning changes. We find the general congruence across models on a median root age for the Dravidian language family of around 4000–4500 years ago including similar 95% HDP ranges supportive of a positive evaluation of the dating results. Nevertheless, the uncertainty on the root age is large, especially for the best-fitting analyses featuring a relaxed clock. Therefore, we cannot exclude the possibility that the root of the Dravidian language family is 6000 or 6500 years old.

## 4. Discussion

Our best-supported model of evolution (the relaxed covarion analysis with relative mutation rates estimated; figure 3) returns a main two-way split between South I (Badga, Toda, Betta Kurumba, Kota, Malayalam, Tamil, Kannada, Tulu, Kodava, Yeruva) and South II–Central–North (Gondi, Kolami, Koya, Telugu, Kuwi, Ollari Gadba, Parji, Brahui, Kurukh, Malto). This main split is also found in a less constrained analysis using the same model of evolution. However, a more constrained analysis, restricting South I and South II to be sisters, finds a different higher-order topology. The best-supported model puts the origin and first diversification of the Dravidian languages at approximately 4500 years ago, a result that matches well with earlier archaeological findings, including inferences made regarding agricultural practices. Below we discuss our subgrouping and dating inference results in the light of previous literature on the Dravidian family.

### 4.1. Higher-order subgrouping

The relationships between the four Dravidian subgroups (North, Central, South II and South I) have been subject to considerable research, but are far from resolved because still little is known on some of the smaller Dravidian languages. While Krishnamurti [7, pp. 492–493] and Southworth [8, p. 235] both group South I and South II together, McAlpin [63, p. 21] finds any attempt of higher structuring of the language groups on the level below the four-part division premature. While there certainly are connections between South II and Central [8, pp. 233–236], Krishnamurti [7, pp. 492--493] shies away from positing a Central + South I + II group as there is little evidence for such a proto-language. Only when we restrain the tree topology, do we find the topology featured in Krishnamurti [7, p. 493] and Fuller [61, p. 200] (as based on [62]), with the North group splitting off first, and South I + South II diverging last. This is not necessarily surprising, as the subgrouping by Krishnamurti [7, pp. 492--493] is based on a small number of phonological and morphological innovations, whereas our subgrouping is based on a Bayesian analysis of cognate-coded material. Rama & Kolachina [21], in an exploratory distance-based analysis of both lexical and phonological/morphological datasets, found that the latter returned the four subgroups very neatly [21, pp. 147–148], while lexical data did not. Given the low posterior support for the Central–North and South II–Central–North groups as presented in figure 3, combined with high support for individual subgroups in the unconstrained analysis (figure 4), we find that the current dataset has low resolution for the higher-order subgrouping, despite recovering the four subgroups with reasonable confidence. While these results could be affected by our failure to identify loan words, the methods we use here are robust to moderate levels of borrowing—Greenhill *et al*. [64] suggest that greater than 15% of the data every 1000 years may be problematic—and it is unlikely that unidentified borrowing is affecting the timeline and topology substantially.

### 4.2. Lower subgrouping

The best-fitting analysis presented in figure 3 returns four subgroups that can be related to the classification by Krishnamurti [7, p. 21] (electronic supplementary material, figure S1): South I, South II, Central and North. The relations of the North languages are clear and in line with previous research; however, the other groups demand further attention. In this discussion, we compare our results to the classifications by Krishnamurti [7, p. 21] and Hammarström *et al*. [1], but also to a large extent draw on grammars and other sources on individual languages cited in the electronic supplementary material, §6.

The South I subgroup, aside from Tulu that splits off first, splits into two smaller groups, one containing Malayalam, Tamil, Kannada, Kodava and Yeruva, the second containing Badga, Toda, Betta Kurumba and Kota. Out of these groupings, the close connection between Tamil and Malayalam is undisputed (it is found in 100% of the posterior tree sample, figure 3). Our best-supported trees pattern differently to Krishnamurti's [7, p. 21] family tree, but we note that Krishnamurti is also unsure about the position of many of these languages (see also [65, p. 14]).

First, Kodava and Yeruva are sisters in our tree set, but belong to different South I subgroups in previous accounts. Hammarström *et al*. [1] give Kodava as most closely related to Tamil and Malayalam, and Yeruva as a Kannadoid language, but see also Bhattacharya [66, p. 32], Luiz [67, p. 27] and Mallikarjun [18, p. 46] for partly conflicting classifications. The reason that we find them as sisters in our tree set is probably due to the fact that Yeruva speakers, especially men, are bilingual in Kodava, a local lingua franca [18, pp. 47–49].

Then, the second South I subgroup, formed by Badga, Toda, Betta Kurumba and Kota. These are four of perhaps 16 small languages spoken in the Nilgiri Hills and their slopes, with Badga, Toda, Kota, and Ālu and Pālu Kurumba speakers living in especially close and long-lasting (over 2000 years) contact [68,69]. The subgrouping of these languages is different across sources (see [1], [7, p. 21], [68, pp. 497, 499, 523], [70, pp. 327–328]). Importantly, Badga is a mixed language, probably once a dialect of Kannada, but has since entered in a diffusional relationship with Ālu Kurumba (primarily), Toda and Kota [71, pp. 42–54], [19]. Previous work indicates that Kota and Toda differ to a large extent from the other South I languages, while their relationship remains unclear [72, pp. 1, 49–50]. It is unclear why in our best-supported tree Betta Kurumba is sister to Kota. Betta Kurumba is located geographically on the outskirts of the cluster containing Toda, Kota, Badga, and Ālu and Pālu Kurumba [68]. Its closest affiliations to other South I languages are unclear, with Zvelebil [68, p. 499] calling it a Kannada dialect, and Upadhyaya [70, pp. 327–328] claiming that Betta Kurumba and Kodava are sisters (echoed in [7, p. 21]).

The current results detect some mixing of the South II and Central branch, which we discuss together. Kolami, a Central language, groups with the South II languages, and when we leave South II unconstrained (see discussion of figure 4), the South II language Kuwi becomes sister to Central Parji and Ollari Gadba. This can be accounted for by an unfortunate lack of data for the Kuwi, Ollari Gadba and Parji languages. Between 36 and 44 comparative concepts are missing, constituting over one-third of the dataset (see §3.1 and table 1). There is enough data to infer their relationship in the Central–South II subgroup overall, but our analysis cannot place them with more certainty within the subgroup itself.

The cluster of Kolami, Gondi, Koya and Telugu does merit further investigation. Kolami is a Central language, found to be most closely related to Ollari Gadba and Parji. However, it has borrowed heavily from Telugu [73, pp. 146–157], [7, p. 26]. We checked those cognate sets that constituted isoglosses for Kolami, Telugu and other South II languages to make sure we had not included obvious Telugu–Kolami loans. They are difficult to identify, but we are confident we have removed them (see electronic supplementary material, §5). A similar explanation can be given for the placement of Koya as a sister to Telugu, rather than Gondi, where it is placed by Hammarström *et al*. [1]. Koya has traditionally been called a dialect of Gondi ([16, pp. 3–4], on Gondi, see [3]). Our sample of Gondi is from Northern Gondi spoken in Madhya Pradesh, whereas Koya is spoken in the Telugu heartland, and has been influenced by Telugu in various ways [16, pp. 4–5], [7, pp. 122, 242, 356]. Our classification of Koya as a sister to Telugu is a result of Telugu influence on Koya as well as the non-geographical adjacency of our sample of Gondi and Koya.

The placement of four language pairs (Kolami–Telugu, Koya–Telugu, Yeruva–Kodava and Badga–Toda–Kota–Betta Kurumba) in our phylogenies may perhaps reflect geographical proximity rather than genealogical descent. This is perhaps an unexpected result, because the Swadesh basic vocabulary list that was used for lexical data collection has been shown to be mostly resistant to borrowing [20]. However, we believe that the topology identified by the current analysis deserves more investigation, especially because it captures certain groupings that have been discussed in the literature, but which have not been captured by traditional expert classifications such as Krishnamurti [7, p. 21] and Hammarström *et al*. [1]. The extent of multi-lingualism and resulting contact also mentioned by Krishnamurti [7, pp. 499–500] regarding the Central languages demands further attention in all of the Dravidian language family. The strength of the approach here is that larger datasets reveal new, targeted directions for investigating contact.

### 4.3. Dating

The age of the tree set of the highest ranking relaxed covarion model of cognate evolution corresponds well with the time depth of the Dravidian language family proposed in earlier literature. We find that the root of the tree has a mean of 4650 years ago (median 4433), thus indicating that the ancestor of all Dravidian languages, Proto-Dravidian, may have been spoken around 4500 years ago. In earlier research, Krishnamurti [7, p. 501] associates Proto-Dravidian with the Indus civilization, places it ‘around the early part of the third millennium’, and adds that even if the Indus valley supposition is incorrect, that date is necessary to account for subsequent developments. Although the mean and median of the best-supported tree set (as well as all other analyses except for the stochastic Dollo) match Krishnamurti's [7, p. 501] timing well, the 95% HPD intervals on the root age range from approximately 3000–6500 years ago. Therefore, we cannot exclude the possibility that the root of the Dravidian language family is significantly older than 4500 years. Contrary to other work by Pagel *et al*. [74], our findings suggest this younger age rather than their Proto-Dravidian estimate of around 13 000 years ago. Future work should investigate the disparity in clock rates here.

Southworth [8, pp. 249–250], on the basis of archaeological findings, linguistic evidence and reconstructed lexicon that at least partly matches the archaeological record, agrees with Krishnamurti on the timing (but not on the homeland, which he places at the lower Godavari basin). Southworth [8, pp. 249–250] places the diversification of the North, Central and South branches in the time period of 4500–4000 years ago, coinciding with the beginnings of the Southern Neolithic complex. Subsequently, he places the expansion of the South I and South II between 4000 and 3000 years ago, coinciding with the expansion of the Southern Neolithic. The dispersal of North, Central and South and later dispersal of South I and South II also matches with the timing in our best-supported trees, notwithstanding that the splitting of our subgroups is binary rather than ternary. The split between South I and the other groups is as ancient as the root of the tree and thus located approximately 4500 years ago. The South I and South II languages start diverging between 3000 and 2500 years ago, which is a little bit later than the timeframe Southworth [8, pp. 249–250] discusses for the expansion of the Southern Neolithic. When the analysis is constrained so that South I and South II form a clade (see the maximum credibility tree in figure 5), the timing of the Southern Neolithic expansion matches the tree structure a bit better, with South II starting to diverge within Southworth's [8, pp. 249–250] timeframe of 4000–3000 years ago.

It is possible to find external corroborating evidence for the dating of the Dravidian dispersals, and in turn, for the calibration points used in the current analysis, by matching language groups with archaeological finds and agricultural practices. Southworth [8, pp. 250–256] tentatively connects South I and South II Dravidian groups with phase 3 (3800–3200 years ago) of the Southern Neolithic archaeological complex. His main concern is that reconstructed vocabulary terms from the South I and II subgroups suggest a society that is advanced beyond anything that has to date been found in the archaeological record of peninsular India, and thus whether there is a mismatch between linguistics and archaeology. Fuller [61, pp. 200–201] suggests that the ancestors of the Central, South I and South II may have spread with the locally developed agricultural economy. He lists crop terms from the South Indian Neolithic native crop package that can be reconstructed to Proto-Central-South, including horsegram, mungbean, urd, pigeonpea and various wild fruits. In addition to these, Fuller [61, p. 203] lists a large number of crops whose terms are reconstructed for Proto-South, including wheat, lentils and cotton. On the basis of reconstructed crop vocabulary and archaeological attestations of crops, Fuller [61, p. 208] tentatively places the differentiation of the Dravidian languages around 6000 years ago, one millennium earlier than Krishnamurti [7] and Southworth [8]. Southworth [75] is a comprehensive analysis of reconstructed crop terms and attestations of the corresponding crops in two phases of the Southern Neolithic archaeological complex. His argument is the same as that of Fuller [61], but importantly, Southworth [75, §5.2] adds to Fuller [61] the rejection of a split between South and Central Dravidian, treating them as a single branch due to early and continued borrowing of words. The diversification of the South I, South II and Central groups in our results is slightly too late to match the start of the spread of the locally developed agricultural economy between 3800 and 3200.

## 5. Conclusion

The history of the Dravidian language family, albeit a small family, is relevant to anyone interested in the history of Eurasia. The current analysis points towards complex patterns of language descent and subsequent long-term contact between languages rather than straightforwardly supporting the well-known reference family tree by Krishnamurti [7, p. 21]. Such diachronic patterns might apply in other small language families as well, making the study of Dravidian relevant for all of historical linguistics. The relationships between the Dravidian languages had previously not all been described to satisfaction, and as this analysis also makes clear, more data on particularly the smaller languages, such as the Gondi dialects, are needed to tease apart descent from contact. This is especially important in the light of the location of the Dravidian language family on the crossroads of ancient population movements into and through South Asia. We propose that our best-supported analysis should be considered the current best estimate of Dravidian genealogical relations, including topology and chronology.

## Supplementary Material

SI_2017_09_29.docx

## Supplementary Material

drav_cov_est_ucln_yule.xml

## Supplementary Material

drav_cov_est_ucln_yule_no_burnin.trees.zip

## Supplementary Material

SI_robustness_cognate_coding.zip

## Supplementary Material

drav_cov_est_ucln_yule.mcct.trees

## Data accessibility

The complete database of cognate data listing coding sources is available on Zenodo and citable as Kolipakam *et al*. [36] (see references, http://doi.org/10.5281/zenodo.1181715). The BEAST 2 xml file for the best-supported analysis, which includes the nexus file containing the binary cognate data on which the phylogenetic analysis is based, as well as further electronic supplementary material, is available through RSOS. For information on BEAST 2, see the website (www.beast2.org), which lists further documentation and resources.
