# Supplementary material for: A Bayesian phylogenetic study of the Dravidian language family
Source: R Soc Open Sci. 2018 Mar 21;5(3):171504. doi: 10.1098/rsos.171504 (PMC5882685; doi:10.1098/rsos.171504)
Supplement: SI_2017_09_29.docx [file rsos171504supp1.pdf]

Supplementary Information

A Bayesian phylogenetic study of the Dravidian language family

Vishnupriya Kolipakam, Fiona M. Jordan, Michael Dunn, Simon J. Greenhill, Remco Bouckaert, Russell D. Gray, Annemarie Verkerk

Royal Society Open Science

Section 1. Calibration points

1. The age of the entire language family. This was controlled with a uniform prior with a lower bound of 0 years, and an upper bound of 10.000 years. Southworth (2005: 40) puts the age of the Dravidian language family at 3000 BCE. Fuller (2007: 429) places the divergence of the Central subgroup around 2000 BCE. There is no indication that this family is much older than 5000 years, so we implemented this prior in order to restrain the search within a 10.000 ya years window.
2. Monophylethic constraint on the North subgroup (involving Brahui, Kurukh, Malto). There are two phonological innovation and four morphological innovations supporting this group (Krishnamurti 2003: 500).
3. Monophylethic constraint on the South I subgroup (involving Badga, Betta Kurumba, Kannada, Kodava, Kota, Malayalam, Tamil, Tulu, Toda, Yeruva). There are one phonological innovations and four morphological innovations supporting this group, with many other isoglosses capturing large subsets (Krishnamurti 2003: 498). This calibration was implemented with a lower bound of 2250 ya, such that this subgroup cannot be younger than 2250 years, as Tamil was attested first in 254 BCE. Steever (1998: 6): "Tamil […] is first recorded in a lithic inscription in a form of Ashokan Brahmi script which is dated to c. 254 BCE."
4. Monophylethic constraint on the South II subgroup (involving Gondi, Koya, Kuwi, Telugu). There are two phonological innovation and five morphological innovations supporting this group (Krishnamurti 2003: 499).
5. The divergence of Brahui. Krishnamurti (2003: 491): "The misleading time depth [of Brahui, ed.] is caused by loss of many cognates in Brahui because of heavy borrowing from Balochi and Indo-Aryan. However, in terms of shared phonological and morphological innovations, it could not have been separated for more than a thousand years or so from Kurukh–Malto." See also Elfenbein (1998: 389). Krishnamurti (2003: 141-142) discusses a sound change from Proto-Dravidian *w > b shared in all three North languages which "is likely to have occurred under the influence of the Magadhan languages of eastern India (Bengal and Bihar)" Krishnamurti (2003: 142). The Eastern Indo-Aryan languages, including Bengali and Bihari, originate in Māghadi Apabhraṅśa, one of several Middle Indo-Aryan varieties (Chatterjee 1926: 21). These are sparsely attested in written records but reconstructed to have been used between 600 BCE and 1000 CE, with Māghadi Apabhraṅśa mentioned specifically in the 200-600 CE time period (Chatterjee 1926: 18-19). This gives an earliest date of the unity of the North subgroup around 200-600 CE, where contact with Māghadi Apabhraṅśa or the languages descending from it could first have taken place. We therefore place a lower bound of 2250 ya on the branch leading to Brahui, as it certainly cannot be older than that.
6. The divergence of Malayalam. Steever (1998: 6): "Between 800 and 1200 CE the western dialects of Tamil, geographically separated from the others by the Western Ghats, developed into Malayalam. The Vaẓappaḷḷi inscription, the

first record of the language, dates to *c*. 830 CE." See also Krishnamurti (2003: 22). This calibration was implemented as a normal probability distribution with a mean of 1000 years ago, a standard deviation of 50, truncated to 800-1200 years ago.

7. The divergence of Telugu. Steever (1998: 4) dates the first Telugu inscription to 620 CE. Krishnamurti (2003: 23): "The first Telugu inscription is dated 575 AD from Erraguḍipāḍu of the Kaḍapa district by a prince of the Cōḍa dynasty". This calibration was implemented as a lower bound of 1400 years ago, as Telugu cannot be younger than that.
8. The divergence of Kannada-Tulu. Krishnamurti (2003: 23) and Steever (1998: 4) date the first Kannada inscription to 450 CE. Krishnamurti (2003: 23): "Kannaḍa […] the first inscription is dated 450 AD by Kadamba Kākutstha Varma from Halmiḍi, Belur Taluq, Mysore district;". Bhat (1998: 159): "The earliest record of Tulu is an inscription dated to the fifteenth century CE." However, we know that Tulu is much older, because it is arguably the first language to split off from the South I group (Subrahmanyam 1968, Rao 1982, Bhat 1998: 160, Steever 1998b: 8). Hence, we placed the ancestor of Kannada-Tulu just after the first Kannada inscriptions were attested, around 700 CE. This calibration was implemented as a lower bound of the ancestor of Kannada and Tulu of 1300 years ago, but Kannada and Tulu were not required to be monophyletic.

Section 2. Reference trees of the Dravidian language family

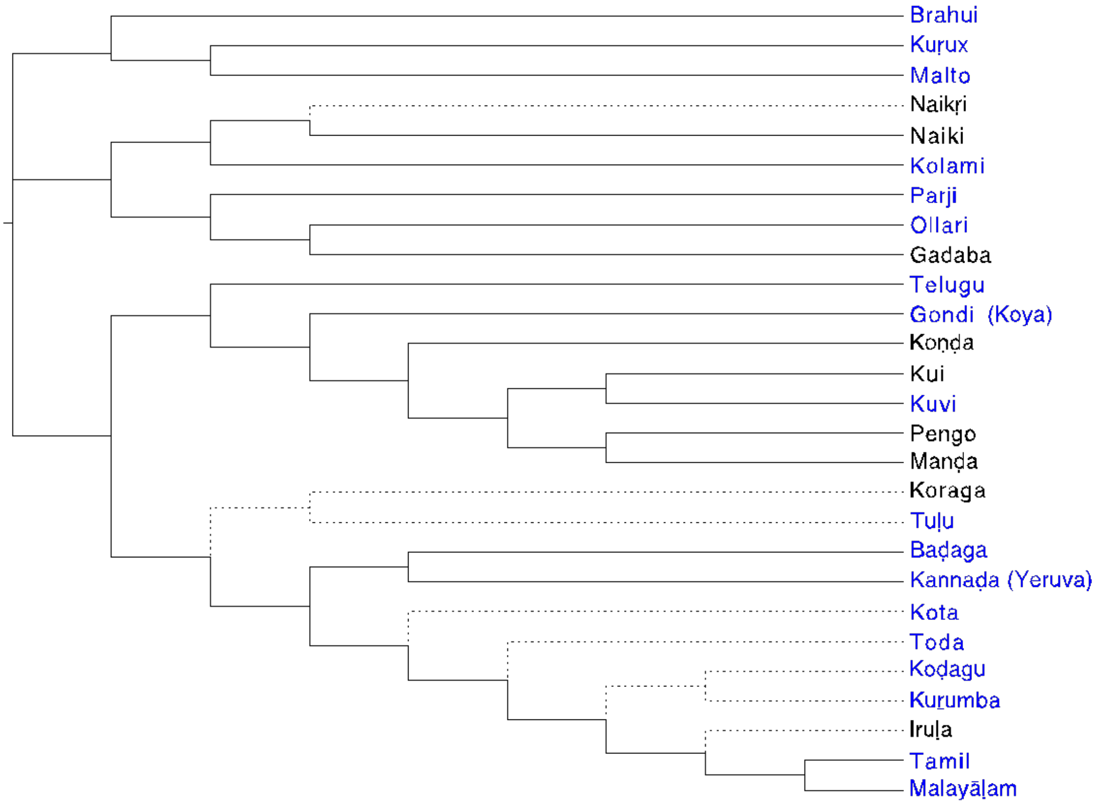

Figure 1: Family tree of the Dravidian languages by Krishnamurti (2003: 21). The dashed tree branches reflect uncertainty with regard to that language's position within the subgroup remarked upon by Krishnamurti (2003). The languages of the current sample are marked in blue, with two dialects not included by Krishnamurti (2003) added in brackets.

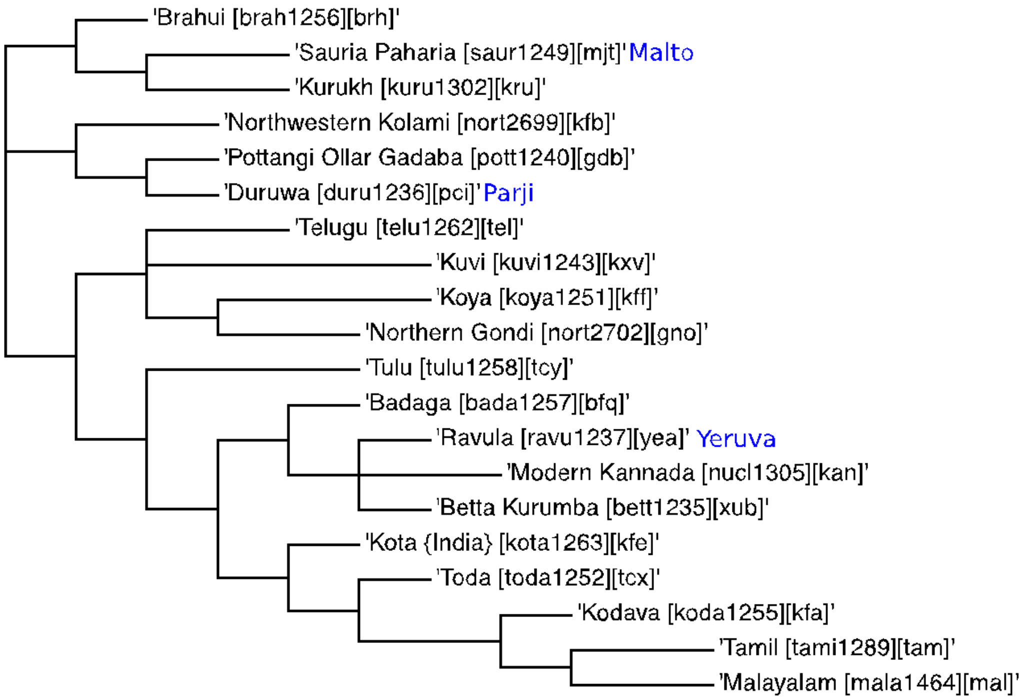

Figure 2: Glottolog expert classification tree for the current sample of Dravidian languages (Hammarström et al. 2016). In blue, language names as used in the current paper that differ from the names used in Glottolog.

Section 3. NeighborNet network

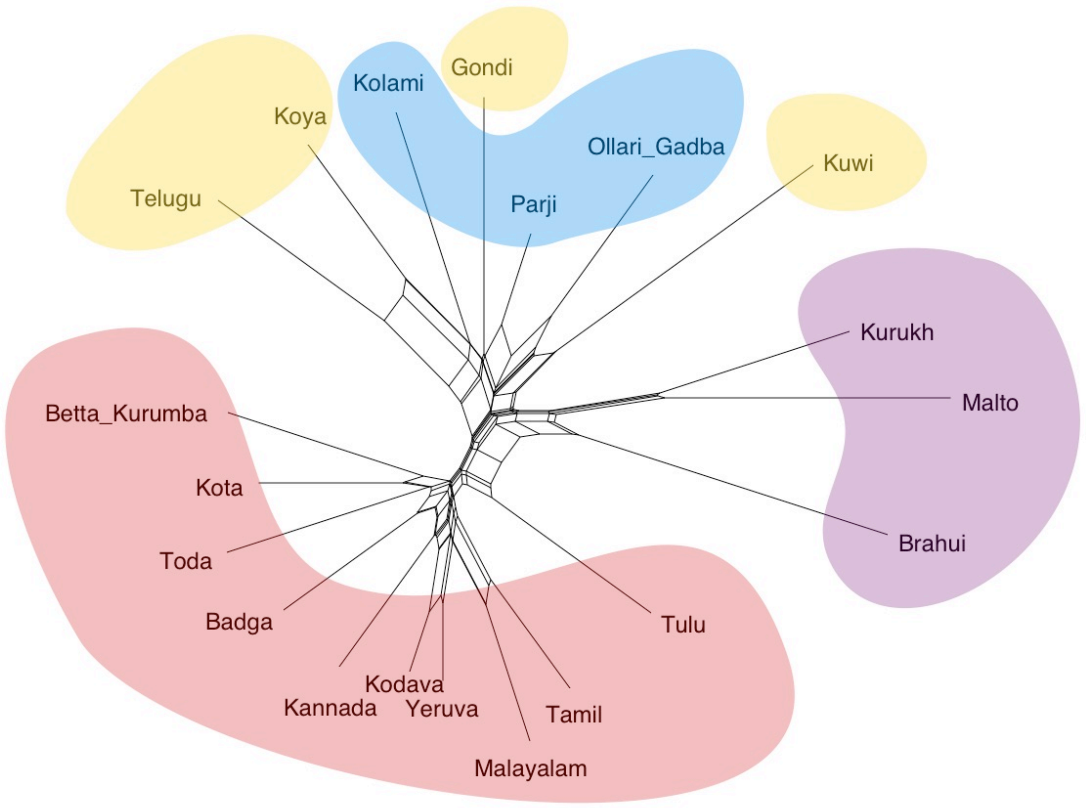

Figure 3. A NeighborNet visualization of lexical differences, obtained by *SplitsTree v.4.14.4*. The NeighborNet network identifies three groups, going in clockwise direction starting from Telugu in the upperleft corner: South II+Central (Telugu, Koya, Kolami, Gondi, Parji, Ollari Gadba, and Kuwi), North (Kurukh, Malto, and Brahui), and South I (Tulu, Tamil, Malayalam, Yeruva, Kodava, Kannada, Badga, Toda, Kota, and Betta Kurumba). Colour coding gives subgroup affiliation, red = South I; blue = Central; purple = North; yellow = South II. Identical to Figure 2 in paper, repeated here for convenience in interpreting the Delta scores in Table 1.

Table 1. Delta scores for the NeighborNet network presented in Figure 3.

**Language**	**Delta score**	**Q-residual**
Brahui	0.28	0.0063
Malto	0.25	0.0051
Kurukh	0.24	0.0046
Ollari_Gadba	0.34	0.0065
Parji	0.31	0.0076
Kolami	0.28	0.0065
Kuwi	0.31	0.0067
Gondi	0.30	0.0069
Koya	0.26	0.0068
Telugu	0.37	0.0132
Tamil	0.30	0.0056
Malayalam	0.29	0.0093
Kannada	0.35	0.0086
Kodava	0.27	0.0059
Tulu	0.35	0.0065
Yeruva	0.28	0.0060
Toda	0.33	0.0060
Kota	0.34	0.0068
Badaga	0.32	0.0055
Betta Kurumba	0.32	0.0047
**mean**	0.30	0.0069

Section 4. Additional Figure and Table for Results

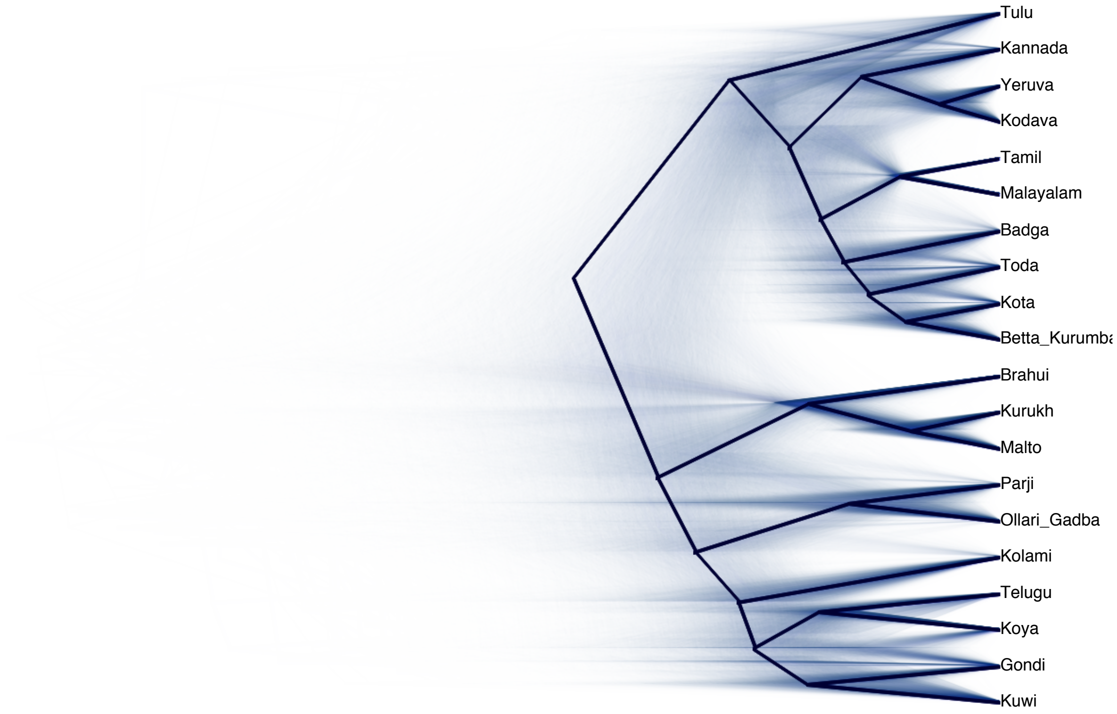

Figure 4. A DensiTree plot (Bouckaert and Heled 2014) of the highest scoring model, the relaxed covarion model with individual mutation rates estimated.

Table 2. Age of the Dravidian language family (tree height), with age in years from the present. Models of evolution are ordered by marginal Lh. See Figure 3 and "Model of evolution" in section 3.2 above for explanations regarding the model, mutation rates, and clock setting used in different analyses.

**Analysis**	**Marginal Log Likelihood**	**Mean Tree Height**	**Median Tree Height**	**Lower Bound 95% HPD Interval**	**Upper Bound 95% HPD Interval**
cov-est-relax	-4128	4650	4433	2812	7063
ctmc-est-relax	-4131	4430	4221	2686	6602
ctmc4g-est-relax	-4136	4559	4521	3761	5434
cov-fixed-relax	-4147	4300	4112	2697	6269
ctmc4g-fixed-relax	-4153	4258	4086	2746	6243
cov-est-strict	-4182	4619	4582	3778	5492
ctmc-est-strict	-4185	4446	4413	3710	5301
ctmc4g-est-strict	-4191	4474	4251	2695	6686
ctmc-fixed-relax	-4234	3943	3822	2811	5317
sdollo-est-relax	-4540	9617	9717	8913	9999

Section 5. Cognate sets shared almost exclusively by Kolami, Telugu, and other South II languages.

Table 3. Sixteen cognate sets present in Kolami, Telugu, and possibly other South II languages, but not (or rarely) in Central, North, or South I languages.

**language**	**1**	**2**	**3**	**4**	**5**	**6**	**7**	**8**	**9**	**10**	**11**	**12**	**13**	**14**	**15**	**16**
Kolami	1	1	1	1	1	1	1	1	1	1	1	1	1	1	1	1
Telugu	1	1	1	1	1	1	1	1	1	1	1	1	1	1	1	1
Malto	0	0	0	0	0	0	0	0	0	0	0	0	0	0	0	0
Kurukh	0	0	0	0	0	0	0	0	0	1	0	0	0	0	0	0
Ollari_Gadba	0	?	0	?	?	0	0	0	?	0	0	0	?	0	0	?
Parji	0	1	?	0	?	0	0	?	?	1	1	?	0	1	0	1
Gondi	1	1	1	1	0	0	0	1	0	1	1	1	1	1	1	0
Kuwi	0	?	?	0	?	?	0	0	?	?	1	0	0	?	0	0
Koya	1	1	1	1	0	1	1	1	1	1	1	1	1	1	1	1
Kannada	0	0	0	0	0	0	0	0	0	0	0	0	0	0	1	0
Badga	0	0	?	0	0	?	0	?	0	0	0	0	0	0	0	0
Kodava	0	0	0	0	0	0	0	0	0	0	0	0	0	0	0	0
Yeruva	0	0	0	0	0	0	0	0	0	0	0	0	0	0	0	0
Malayalam	0	0	0	0	0	0	0	0	0	0	0	0	0	0	0	0
Tamil	0	0	0	0	0	0	0	0	0	1	0	0	0	0	0	0
Betta_Kurumba	0	0	0	0	0	0	0	0	0	0	0	1	0	0	0	0
Kota	0	0	0	0	0	0	0	0	0	0	0	1	0	0	?	0
Toda	0	0	0	0	0	0	0	0	0	0	0	0	0	0	0	0
Tulu	0	0	?	0	0	?	0	0	0	1	0	0	0	0	0	0

Table 4. The Telugu and Kolami reflexes and etymological notes regarding the sixteen cognate sets in Table 3.

**meaning**	**Kolami**	**Telugu**	**Emeneau (1955)**	**DEDR**	**Borrowing?**
leaf	aːk	aːku	-	335	Probably not
bone	bokːa	bokːa	104	4528	Probably not
heart	gunɖe	guɳɖa	-	1693	Probably not
red	jeruɖi	eɾupu	2098	865	Probably not
not	kaːdu	kaːdu	-	-	Could be
liver	kaleja	kaːlejamu	-	-	?
new	kota	kotːa	-	2149 ?	Probably not
bird	piʈːa	pitːa	3112	4154	Could be
long	poɖaːm	poɖaʋu	703	4484	Probably not
small	sinːa	tʃinːa	834	2594	Probably not
star	sukːa	tʃukːa	868	2646	Probably not
sun	poddu	poddu	699	4559	Could be
white	telːuɖi	telupu	912	3433	Probably not
tail	toːka	toːka	942	3538	Probably not
sand	usuka	isuka	2346	575	Could be
rain	ʋaːna	ʋaːna	1009	5381	Probably not

In Table 4, the column entitled ‘Emeneau (1955)’ refers to his grammar of Kolami, which has a large lexicon from page 175 onwards. The column entitled ‘DEDR’ refers to Burrow and Emeneau (1984), accessed online at http://dsal.uchicago.edu/dictionaries/burrow/

As can be observed in Table 4, we identified four cognate sets that could potentially be borrowings from Telugu into Kolami. We removed these from the nexus file and build a new NeighborNet, which is presented in Figure 2. The position of Kolami with respect to Telugu does not change. Hence, we believe that even if these are indeed borrowings, our results regarding the placement of Kolami with respect to the South II languages still stand.

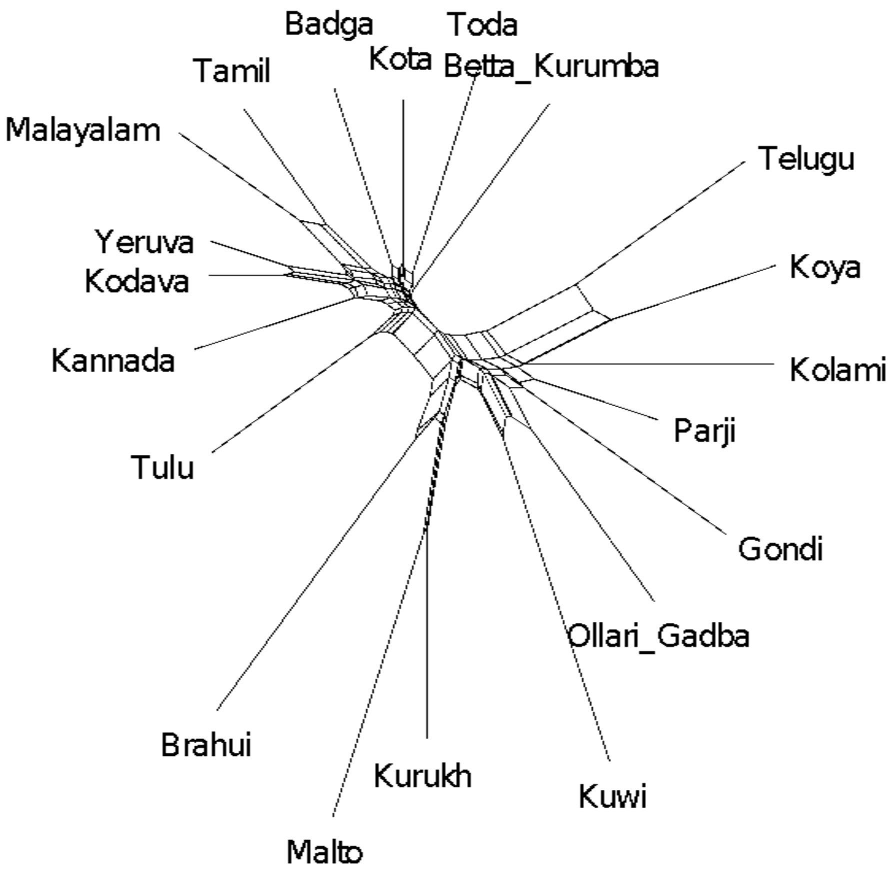

Figure 5. A NeighborNet visualization of lexical differences excluding the relevant cognate sets for *not*, *bird*, *sun*, and *sand*. Obtained using SplitsTree (Huson and Bryant 2006).

Section 6. A literature review of the languages in the sample, detailing information found on multilingualism and accounts of relationships to other Dravidian languages

Table 5. Multilingualism in the smaller languages of the current sample based on Lewis et al. 2016 and other sources, see below. Other languages spoken are listed in order of relevance. Their isocodes and family affiliation is given in brackets (D: Dravidian, IE: Indo-European, M: Munda, Austroasiatic).

**language community**	**other languages spoken**
Brahui (brh)	Western Balochi (bgn, IE)
Malto (mjt)	Santali (sat, M), Hindi (hin, IE), Bengali (ben, IE)
Kurukh (kru)	Mundari (unr, M), Kharia (khr, M), Hindi (hin, IE), Sadri (sck, IE), English (eng, IE)
Ollari Gadba (gdb)	Desiya (dso, IE), Telugu (tel, D)
Parji (pci)	Halbi (hlb, IE), Hindi (hin, IE), Odia (ory, IE), Bhatri (bgw, IE)
Kolami (kfb)	Marathi (mar, IE), Gondi (gno, D), Telugu (tel, D)
Kuwi (kxv)	Desiya (dso, IE), Telugu (tel, D)
Gondi (gno)	Hindi (hin, IE), Marathi (mar, IE)
Koya (kff)	Telugu (tel, D), Hindi (hin, IE)
Kodava (kfa)	Kannada (kan, D), English (eng, IE), Malayalam (mal, D)
Tulu (tcy)	English (eng, IE), Hindi (hin, IE), Kannada (kan, D), Marathi (mar, IE)
Yeruva (yea)	Kodava (kfa, D), Kannada (kan, D), Malayalam (mal, D)
Toda (tcx)	Tamil (tam, D)
Kota (kfe)	Tamil (tam, D), Badaga (bfq, D), Kannada (kan, D), English (eng, IE)
Badaga (bfq)	Tamil (tam, D), English (eng, IE), Kannada (kan, D)
Betta Kurumba (xub)	Tamil (tam, D)

North languages

Brahui (brh)

Lewis et al. (2016):

1. numbers of speakers: “2,210,000 in Pakistan (2011). Total users in all countries: 2,430,000.”
2. sociolinguistic situation: “Also use Western Balochi [bgn].”

Elfenbein (1998):

p. 388-389: “There are approximately 700,000 Brahui tribesmen, mainly in Pakistani Baluchistan and in Afghanistan. Of these, approximately 100,000 are primary speakers of Brahui, mainly in Pakistan; perhaps 300,000 are secondary speakers of Brahui in Pakistan and Afghanistan. Among the secondary speakers, the primary language is the Iranian language, Balochi. Further, most primary speakers of Brahui speak some Balochi as well. But fully 300,000 Brahui tribesmen speak no Brahui at all.”

p. 389-390: “Language use among the approximately 300,000 secondary speakers of Brahui is extraordinary. Bilaterally bilingual in two genetically unrelated languages, Brahui and Balochi, the speakers use both languages every day, but consciously keep

them apart. Even so, the mutual influence of the two languages on each other is evident. An account of the linguistic sociology of this group appears in Encyclopedia Iranica, 438ff."

Malto (mjt)

Lewis et al. (2016):

1. numbers of speakers: "54,000 in India (Bhaskararao 2006). Total users in all countries: 61,000."

Steever (1998):

p. 359: "Malto is a non-literary language and lacks official status. The earliest records of the language data to the last century. It has come into contact with neighbouring Indo-Aryan languages such as Hindi, Oriya and Bengali."

p. 385: "As noted earlier, Malto has three dialects: Sawriya with about 54,000 speakers, Malpaharia with 40,000 speakers and Kumarbhag with 12,500 speakers."

Das (1973):

p. 1: "Malto, a Dravidian language, is spoken by one of the primitive races of India who call themselves *maler* 'men.'"

p. 5: "The *maler* are a bilingual community: they speak Malto only in their villages and speak either Santali or a dialect of Hindi or Bengali in their intercourse with the outer world. And as a result the process of borrowing is almost continuous in Malto and the people frequently use Hindi or Bengali words in their every day conversations. A larger number of religious words, for example, have come from Indo-Aryan as well as from English, thanks to the missionaries."

p. 6: "I have met several *maler* who speak Malto only in home and their children do not know any Malto. Those who live in the plains where one has to come into contact with the Santals and the Biharis they find that their children seldom use Malto. The Government of Bihar has established several schools, for the paha̱riyas, for example one in Banjhi and one in Hiranpur, where they receive free education. Unfortunately there is no arrangement for teaching Malto to the *maler*. The Government is trying to improve their economic and social conditions but it is entirely indifferent to their language which is sure to perish and to be replaced by Hindi."

Kurukh (kru)

Lewis et al. (2016):

1. numbers of speakers: "1,750,000 in India (2001 census). Total users in all countries: 1,804,200."

Mishra (1991):

p. 26-27: "The percentage of Kurux speaking Oraons to their total population is highest in the Mandar, Kisko, Chanho, Kuru, Lohardagga, Bisunpur, Dumri, Chainpur and Raidih community development Blocks. It is the mother tongue of more than 90% of the Oraon population in those Blocks which constitute the most compact area of Kurux language in the district. In the Blocks of Gumla, Sisai, Varno, Bero, Kanke, Bhandra and Ratu between 70% and 90% of the Oraons speak Kurux as their mother tongue. In the Blocks of Karra, Kurdeg, Thethaitanagar and Jaldega, between 51% and 79% of the Oraons speak Kurux as their mother tongue. Although in Basia, Bhundu, Ormanjhi, Bolba, Palkot and Tamar-I Blocks the Oraon population is very large, yet the percentage of Kurux speaking Oraons is only 0.34, 18.20, 0.33, 15.09 and 6.67 respectively in these Blocks. This low percentage may be attributed to the cultural influences of Mundari and Kharia speaking population. […]

In the areas dominated by Mundari and Kharia population the uneducated persons,

especially women, use Mundari and Kharia languages while dealing with the people of those communities. Educated persons use Hindi and Sadri in urban areas and Sadri in rural areas. The urban Oraons consider that the use of Hindi makes them superior in comparison with other fellow Oraons who cannot speak it. […] The spread of education also necessitated the use of Hindi, Sadri and even English by the Oraons. […]
Although Sadri is the lingua franca of Ranchi district, in sever- al Blocks, the female population is still monolingual. It may be due to the fact that there is less contact with other castes and tribes and less formal education among female Oraons than among male Oraons. On the other hand, the marriage relations of the Oraon females in Basia, Khunti, Bundu and Tamar-I Blocks extend to such villages where other tribes and castes use Sadri as their mother tongue. The long contact with such tribes and castes has negatively influenced the lingual attachment of the Oraons. They have forgotten Kurux and the number of Oraons who give up Kurux is increasing everyday."

Central languages

Ollari Gadba (gdb)
Lewis et al. (2016):
1. numbers of speakers: "15,000 (2002 M. Kurian). 4,000-7,000 in Koraput District, Pottangi block (1995)."
2. sociolinguistic situation: "Positive attitudes. Also use Desiya [dso], Telugu [tel]."

Bhaskararao (1998):
p. 328: "Speakers of Gadaba live in a continuous area that traverses the north of Andhra Pradesh and the southwest of Orissa. […] The earliest systematic study of this language is Bhattacharya (1957); in this work and some others, the language is called Ollari. Burrow and Bhattacharya (1962-3) note that Ollari and Koṇekor Gadaba are two local variants of the same language. While the Census of India does not distinguish between Koṇekor and Gotub Gadaba, the size of the Koṇekor-speaking population is estimated at a few thousand."
p. 352: "The lexicon consists of native and borrowed lexical items. For the dialect that is studied, Telugu seems to be the major source of borrowings though a few lexical items come from Oriya."
p. 354: "The Koṇekor dialect appears to borrow more heavily from Telugu since that is the dominant regional language; similarly, the Ollari dialect leans more towards the Dēsiya dialect of Oriya."

Parji (pci)
Lewis et al. (2016):
1. numbers of speakers: "51,200 (2001 census). Ethnic population: 100,000 (1986). 65% in Bastar, 35% in Koraput."
2. sociolinguistic situation: "Madiya [mrr] speak Duruwa to communicate with the Dhurwa people. Also use Halbi [hlb], Hindi [hin], Odia [ory]. Also use Bhatri [bgw], in northern Bastar District."

Kolami (kfb)
Lewis et al. (2016):
1. numbers of speakers: "122,000 (2001 census)."
2. sociolinguistic situation: "Home, community. Also use Marathi [mar],

Northern Gondi [gno], Telugu [tel].”
Subrahmanyam (1998):
p. 326: “As most Kolams also speak Marathi, some Indo-Aryan features have naturally entered Kolami. […] Kolami has borrowed words from Marathi and Telugu, two major regional languages. While it is often difficult to distinguish native Kolami words from Telugu loans since both are Dravidian, Emeneau (1955) formulated some principles for identifying loans from sister languages. He estimates that of 931 words in the Wardha corpus, 55 per cent are Dravidian, 35 per cent Indo-Aryan and 10 per cent unknown. He further estimates that about 30 per cent of the Dravidian words (213 out of 720) come from Telugu.”
Emeneau (1995):
p. 146: “Upon application of these principles and that involved in proved examples of “exclusively shared innovations” (see 10.11), it will be shown that Kol. had an original history of development independent of Te. but that in more recent times it has borrowed heavily from Te.”
p. 156: “In all, approximately 166 borrowings from Te. have been identified out of the 720 items with Dr. etymologies, i.e., about 23 per cent of the Dr. part of the vocabulary is certainly borrowed from Te.”
p. 157: “A numerical treatment, that would seem fairly well justified, is to assume that the proportion between borrowings and inheritances in these 159 items is the same as in the rest of the items with Dr. etymologies. The latter figures have already been given in 10.20, 28 as 166 borrowings from Te. (23 per cent of the total vocabulary with Dr. etymologies) and 395 items which are not borrowings from Te. (55 per cent). When this proportion (166: 395) is applied to the 159 uncertain items, they are divided into 47 borrowings and 112 nonborrowings. Addition of these figures to the others given 213 borrowings from Te. and 507 items that are not borrowings from Te., or 30 per cent and 70 per cent respectively of the total 720 items with Dr. etymologies.”

South-II languages

Kuwi (kxv)
Lewis et al. (2016):

1. numbers of speakers: “158,000 (2001 census).”

Anand (2002: 570): “The Kuvis are bilinguals in Desia, a variety of Oriya and in Telugu that they generally use among their fellow men of Dravidian ethnics. Besides, a few of them can communicate in standard Oriya, the official language of the state and in Hindi as well.”

Gondi (gno)
Lewis et al. (2016):

1. numbers of speakers: “1,950,000 (1997 BSI). 2,630,000 all Gondi.”
2. sociolinguistic situation: “All ages. Positive attitudes. Also use Hindi [hin], Marathi [mar]. Used as L2 by Northwestern Kolami [kfb].”

Steever (1998):
p. 270: “Goṇḍi belongs to the South-Central branch of Dravidian. It is spoken by perhaps two million people in the central Indian states of Maharashtra (1,300,000), Madhya Pradesh (450,000), Andhra Pradesh (270,000) and Orissa (84,000). Goṇḍi’s extensive dialect variation may be attributed to several factors: it covers a wide geographic area, has no written tradition and lacks official status. ”

Beine (1994):
p. 17: “Krishnamurti (1976: 139-153) moves Gondi from the central Dravidian branch to a new branch called South Dravidian II, which consists of Gondi, along with several other languages that are more usually considered to be central Dravidian. This is an important proposal and by no means uncontroversial, especially as the analysis upon which the change is made has not yet been made available. […] Historical linguists are otherwise fairly united in their placement of the Gondi language in the central Dravidian family.”

Koya (kff)
Lewis et al. (2016):
1. numbers of speakers: “362,000 (2001 census).”
2. sociolinguistic situation: “Some also use Telugu [tel], but proficiency is low.”

Beine (1994):
p. 21: “He indicates that some groups of Koyas still speak Koya (a Gondi dialect), but the majority have lost their language in place of Telugu.”
But it’s unclear what he means with this. He talks about Dorla Koya, but only includes ‘Koya Goti’ in his survey, which is spoken in Nirmal Taluk, Adilabad District, Andhra Pradesh. Code is gni, p. 96.
Tyler (1969):
p. 3: “Along the Godavari River and near major roads the Koyas live in contact with Telugu-speaking castes. To the north, along the Bastar border, they are in contact with various Gondi-speaking tribes and Indo-Aryan speaking Hindi castes. Consequently, most Koya males are bilingual, speaking Koya and Telugu or Koya and one or another Hindi dialect. In more remote areas the majority of females speak only Koya with a minimum of bazaar Telugu, Hindi, or Oriya. Since Koya is a Gondi language, it is mutually intelligible with Hill Maria Gondi in Bastar and Sironcha. Though I have no real evidence, the general pattern seems to be for geographically adjacent Koya and Gondi populations to speak different, but mutually intelligible Gondi dialects. Where these populations are geographically non-contiguous, the dialects are not mutually intelligible. This same pattern probably prevails among all Gondi dialects.”
p. 4: “The language and culture of the Gommun Koyas has been influenced to a great extent by contact with Telugus. The same is true for Lingu and Bāsa Koyas. The former are reported to be Lingayats (Prasad, 1950: 163-164). Little is known about Guṭṭa and Dōrla Koyas, but they are distinguished from Gommu Koyas by the fact that they have been less influenced by Hindi customs and still practice swidden agriculture. They also retain more of the cycle of agricultural rituals associated with swidden agriculture. Gommu Koyas regard Guṭṭa Koyas as being “wilder” and less “civilized”, whereas Guṭṭa Koyas look down on the Gommu Koyas as an inferior group of brawlers and cattle thieves.”

Telugu (tel)
Lewis et al. (2016):

1. numbers of speakers: “73,800,000 in India (2001 census). L2 users: 5,000,000 in India. Total users in all countries: 79,244,300 (as L1: 74,244,300; as L2: 5,000,000).”
2. sociolinguistic situation: “Used as L2 by Bodo Gadaba [gbj], Chenchu [cde], Koya [kff], Kupia [key], Lambadi [lmn], Mudhili Gadaba [gau], Northwestern

Kolami [kfb], Pottangi Ollar Gadaba [gdb], Vaagri Booli [vaa], Waddar [wbq], Yerukula [yeu].”

South-I languages

Tamil (tam)
Lewis et al. (2016):
1. numbers of speakers: “60,700,000 in India (2001 census). L2 users: 8,000,000 in India. Total users in all countries: 75,832,790 (as L1: 67,832,790; as L2: 8,000,000).”
2. sociolinguistic situation: “All also use Kannada [kan] (Gowda 1976). Used as L2 by Alu Kurumba [xua], Attapady Kurumba [pkr], Eravallan [era], Irula [iru], Jennu Kurumba [xuj], Kadar [kej], Kanikkaran [kev], Kannada Kurumba [kfi], Kota [kfe], Malankuravan [mjo], Malasar [ymr], Muthuvan [muv], Paliyan [pcf], Paniya [pcg], Saurashtra [saz], Toda [tcx], Vaagri Booli [vaa].”

Malayalam (mal)
Lewis et al. (2016):
1. numbers of speakers: “33,000,000 in India (2001 census). Total users in all countries: 34,261,600.”
2. sociolinguistic situation: “Cochin Jews in Kerala speak Malayalam. Used as L2 by Allar [all], Aranadan [aaf], Attapady Kurumba [pkr], Irula [iru], Jennu Kurumba [xuj], Kadar [kej], Kalanadi [wkl], Kanikkaran [kev], Kodava [kfa], Kudiya [kfg], Kunduvadi [wku], Kurichiya [kfh], Mala Malasar [ima], Malapandaram [mjp], Malasar [ymr], Malavedan [mjr], Mannan [mjv], Muduga [udg], Mullu Kurumba [kpb], Muthuvan [muv], Paliyan [pcf], Paniya [pcg], Ravula [yea], Thachanadan [thn], Urali [url].”

Kannada (kan)
Lewis et al. (2016):
1. numbers of speakers: “37,700,000 in India (2001 census). L2 users: 9,000,000 in India. Total users in all countries: 46,739,040 (as L1: 37,739,040; as L2: 9,000,000).”
2. sociolinguistic situation: “Used as L2 by Alu Kurumba [xua], Holiya [hoy], Irula [iru], Jennu Kurumba [xuj], Kannada Kurumba [kfi], Kodava [kfa], Kota [kfe], Kudiya [kfg], Lambadi [lmn], Mudu Koraga [vmd], Paniya [pcg], Ravula [yea], Tamil [tam], Tulu [tcy], Vaagri Booli [vaa].”

Kodava (kfa)
Lewis et al. (2016):
1. numbers of speakers: “200,000 (2001). Ethnic population: 200,000. 100,000 in Kodagu District; 100,000 in Karnataka District and major cities.”
2. sociolinguistic situation: “Vigorous. L1 of Airi, Male-Kudiya, Meda, Kembatti, Kapal, Maringi, Heggade, Kavadi, Kolla, Thatta, Koleya, Koyava, Banna, Golla, Kanya, Ganiga, and Malaya. Home, community, religion. Most also use Kannada [kan]. Many also use English [eng]. Also use Malayalam [mal]. Used as L2 by Kudiya [kfg].”

Balakrishnan (1977):
p. x: “The Kodavas are more keen in preserving their customs and traditions. They have a cosmopolitan outlook regarding their language. Kannada is the official

language and all Kodavas have to learn it out of necessity – though there is hardly any chance of two Kodavas conversing in Kannada. During the British rule, English words was a prestigious language and Kodavas took pride in acquiring this language and as a result one can see many English words being borrowed into Kodagu. Though Kannada is the school and official language, it is rather surprising that it is not possible to count by fingers even the number of Kodavas excelled themselves as Kannada scholars whereas the number of those excelled themselves in English is quite remarkable. However, the mother tongue though it is neglected to any extent must be regarded as a mirror reflecting many of the ancient features."

Ebert (1996):

p. 5: "Koḍava is a South Dravidian language spoken in Coorg district by approximately 70.000 people. The language is better known by its Kannada name "Koḍagu". The Koḍava people are bilingual in Kannada, and they claim that their language is a dialect of Kannada. This belief seems to be founded in the fact that Koḍava is written with the Kannada script. Of course Koḍava has borrowed a number of words from its neighbor, but it is more closely related to Tulu and Tamil."

Tulu (tcy)

Lewis et al. (2016):

1. numbers of speakers: "1,720,000 (2001 census)."
2. sociolinguistic situation: "Also use English [eng], Hindi [hin], Kannada [kan], Marathi [mar]. Used as L2 by Korra Koraga [kfd], Kudiya [kfg]."

Bhat (1998):

p. 158: "Tulu (tuḷu) is spoken by more than three million people, half as their mother tongue and half as a second language. […] Tulunad (tuḷunāḍū), where Tulu is traditionally spoken, is geographically and sociolinguistically compact. Its geographic compactness derives from the natural boundaries that enclose it: the rivers Suvarna and Chandragiri form its northern and southern boundaries while the Western Ghats and the Arabian Sea set its eastern and western boundaries. […] The Netravati river divides Tulunad into two nearly equal parts, a division that has produced distinct north and south dialect areas."

p. 159: "One notable distinction between these two areas is the relative prestige accorded to Tulu as a *lingua franca*. In the north Tulu commands high prestige so that even educated people with different mother tongues use it to communicate. In the south, however, it has less prestige and educated people prefer Kannada for mutual communication. Even so, the rise of Tulu in novels, drama, cinema, and political and cultural forums has recently enhanced the language's status in the south, where its use in formal communication is gaining ground. […] The earliest record of Tulu is an inscription dated to the fifteenth century CE."

p. 160: "The prehistory of Tulu, particularly its relation with other Dravidian languages, is disputed. According to Subrahmanyam (1968), Tulu belongs to South Dravidian whereas Rao (1982) places it closer to Central Dravidian. Subrahmanyam does concede that Tulu branched off from Proto-South Dravidian earlier than the other South Dravidian languages."

p. 173: "The debates in the specialist literature generally focus on ways in which Tulu phonology and morphology differ from those of other Dravidian languages. But the most solid conclusion one may draw from many of these arguments is that these differences represent independent innovations within Tulu rather than shared innovations with the South-Central languages. The further question of affiliating Tulu to a specific subgroup requires a greater understanding of the history and synchrony

of languages which are at present only fragmentarily understood. Hence, arguments for subgrouping cannot be considered decisive at our current state of knowledge; as the languages involved come to be known in greater detail and clarity, this issue can be more adequately debated."
Steever (1998: 8): "Tulu appears to share several features with the South-Central (South II, red.) Dravidian languages, so many in fact that some scholars place it in that subgroup. It may well be the first individual language to branch off of Proto-South Dravidian."

Yeruva (yea)
Lewis et al. (2016):

1. numbers of speakers: "26,900 (2007). 25,000 Yerava and 1,900 Adiya. Ethnic population: 47,000 (2007)."
2. sociolinguistic situation: "Vigorous. Home, village, religion. Positive attitudes. Also use Kannada [kan], Malayalam [mal]."

Mallikarjun (1993):
p. 47: "It is to be noted that 1961 Census reports more number of Yeravas as bilinguals in Kannada. It is also to be noted that Yerava females out number Yerava males in bilingualism according to the Census report. But the present investigator has found during his field trips in South Kodagu that more Yeravas are bilinguals in Kodagu rather than in Kannada. He has, however, no means to make a statistical survey to substantiate further this observation. The situation deserves a deeper analysis. In any case, the following hypothetical reasons may be given for the type of statistical information obtained by Census. The first reason is that Yeravas may like to identify themselves with Kannada, because Kannada is the language of education, administration and mass communication in Karnataka including the district of Kodagu. The second reason is that the enumerators might have presented the languages in the order of Kannada, Kodagu, etc., and the Yeravas might have nodded 'yes' to that order."
p. 49: "Within a group of Yeravas, they speak in their tongue only and never use any other language that they may know. The male Yeravas know Kodagu well. While speaking to Kodavas, they invariably speak in Kodagu. However, some of the Kodavas say that they speak with Yeravas in Yerava. But the present investigators observation indicates the following in these contexts. When a Kodava questions or answers to an Yerava, the Kodava picks up the words of Kodagu and suffixes of Yerava and speaks with that combination. And some times he combines Yerava words and Kodagu suffices to speak to an Yerava. All this depends upon his understanding or knowledge of Yerava tongue. One notices a mixed language of Yerava and Kodagu, especially when a Kodava speaks to an Yerava. Next to Kodagu speakers, Yeravas come across Kannada speakers more often, though native Kannada speakers are numerically less in these parts. However, Kannada being a school and state language it is spoken also in the environment of Yeravas. Hence, Yerava menfolk have more acquaintance than the Yerava womenfolk with Kannada speaking environment. (The bilingualism statistics given by 1961 Census gives an opposite picture as already pointed out.) The womenfolk have only a nodding acquaintance with Kannada. Among Yerava men also persons around 45 years and above have very little acquaintance with Kannada. But the people of younger generation, namely those of 20-35 years have a good knowledge of spoken Kannada. However, one notices differences between the Kannada spoken around the young Yerava and the way he speaks Kannada. The shops, hotels and other business establishments around Yeravas

are run by Malayalee Mapillas. Because of mutual dependence, the Yeravas and Malayalees understand each others language to the extent that the transactions demand such an understanding. The Yerava of one group comes across the Yerava of another group in work place, shandy, etc. In such a context, if he desires to communicate, the Yerava uses his own dialect for the purpose. Because of the mutual intelligibility, Yeravas of both the groups do not face any difficulty in understanding each other."
The classification found in Glottolog (Hammarström et al. 2016) suggests Yeruva is most closely related to Kannada, as well as several other smaller languages, including Betta Kurumba. But the source they cite for this, Battacharya (1976), in fact states Yeruva (there called Mala Adiyan, as well as other varieties investigated, are dialects of Malayalam: (p. 32): "These tribal tongues may therefore be designated as separate tribal dialects of Malayalam."

Toda (tcx)
Lewis et al. (2016):

1. numbers of speakers: "1,560 (2001 census)."
2. sociolinguistic situation: "Vigorous. All domains. All ages. Positive attitudes. Also use Tamil [tam]."

Emeneau (1957):
p. 28: "One factor that may seem surely relevant is whether the Toda language is intelligible to speakers of other Dravidian languages. The answer to this question is no. Not even their nearest neighbours, the Kotas and the Badagas, can understand Toda without spending time on learning the language, and in fact no Kotas and very few Badagas ever learn it well. The same probably is true of the other indigenous Nilgiri community, the Kurumbas (we have little linguistic information about them), and is certainly true of the more recent colonies of Tamil and Kannada speakers in the Nilgiris. There is no need even to mention the other, geographically more remote, Dravidian languages. The situation is striking. Todas, Kotas, and Badagas all live, and have lived for centuries, in an area of no more than forty by twenty miles, with their villages sandwiched in among one another in the most intimate fashion, and yet three mutually unintelligible languages are spoken. The diversity fostered by the Hindu caste structure is clearly the operative factor here – incidentally, similar situations are found elsewhere in India; the problem requires much more detailed description and generalized discussion than it has yet received."
p. 49-50: "The chronological implication is clear. We have already seen (§32) that because Toda does not show palatalization of PDr *k- before front vowels, it must have been separate from Tamil before the beginning of the Tamil records. The features of the non-personal nouns just discussed make it necessary to put Toda together with Tamil earlier than this period, and to speak of Toda as closely related to pre-Tamil, with a separation prior to our records of Tamil. […] The problem of the relationship between Toda and Kota must be left for the moment. Too much remains to be worked out about both of them for a solution to the problem to be attempted at this time."
Emeneau (1984):
p. 1: "The Todas are a small community who live on the isolated Nilgiri plateau in South India (now a part of Tamilnadu). They lived there in aboriginal days, that is, prior to the early nineteenth century, in coexistence with Badagas, Kotas, and Kurumbas (and several other jungle communities). The local social organization was a caste-like system in which the Todas were the top-ranking community. […] the

language of the Todas is a member of the Dravidian family. It, like their ethnology, is aberrant and, in its phonological aspects, difficult. […] It is no recognized that Toda (along with its neighbor Kota) is a member of the southern subgroup of the family, and that these two, as a Nilgiri subgroup, are closely related to Tamil (Emeneau 1957, 1967b). To use very modern terminology, the aberrancy of Toda results from disproportionately numerous rules, both early and recent in their ordering, which are not shared by the other South Dravidian languages (or which are scared only to a small extent by Kota)."

Zvelebil (1981):

p. 495: "Before the social disruption of the Nilagiris began more than a century and a half ago upon the arrival of the British and their opening of the area to extensive Indian migration from the plains, the Nilagiri area was the home of a miniature local 'caste system' of four groups – Todas, Badagas, Kotas, Kuṟumbas. It had many of the typical caste-system features - a ranking of the four communities, economic relations of the jajmani type, endogamy, and maintenance of differences and distance. There were no multi-caste, multi-tribal villages or settlements (as there are today) […] The Todas were already in the Nilagiris in A. D. 1117, according to a Kannaḍa inscription, safely dated, which mentions them but no other Nilagiri tribe"

p. 495-496: "The three tribes of the Todas, Kotas, and (Ālu, Pālu) Kuṟumbas regard themselves as being the aboriginal inhabitants of the Nilagiri mountains, and as having been created there together (cf. Emeneau 1938: 101). The three tribes of the Todas, Kotas, and Kuṟumbas, along with the Badaga community, formed the internal Nilagiri system, the inner circle or inner infrastructure, at least since the beginning of the 17th century, till about a hundred years ago. The core of this system was the tribal interrelationship of the 'original' Nilagiri tribes, Todas, Kotas, and Kuṟumbas, who regarded themselves as autochthonous in the area, and have lived in the mountains together for maybe the past 2000 years or more. Around these communities spreads the peripheral tribal ring: the Iṟulas proper in two moieties, Mele Nāḍu and Vĕṭṭe Kāḍu Iṟulas, in the marginal jungles of the lower slopes and valleys; and, in the southern Wynaad area, as well as on the lower western and southwestern slopes, the tribes of the Iṟula-speaking Kasabas; the Jēnu Kuṟumbas Bĕṭṭa Kuṟumbas, Muḷḷu Kuṟumbas, Ūrāḷi Kuṟumbas, Paṇiyas, and Šōlegas. The Ūrāḷi Iṟulas live north of the mountains proper."

p. 497: Zvelebil divides up the 16 Nilagiri languages in a Toda-Kota subgroup, a Tamiloid subgroup, and a Kannadoid subgroup. Betta Kurumba is found in the Kanndadoid subgroup.

p. 523-524: this is not on Toda specifically, but generally on the situation in the Nilagiris: "As one looks at the linguistic map of the larger Nilagiri area and the surrounding linguistics space and considers the type and character of the languages involved one finds on the one hand the relatively sharply delimited large literary languages Tamil, Malayalam, and Kannaḍa, and on the other hand a number of tongues spoken by smaller, non-literate communities. Most of these – with the exception of Badagu (most likely a Kannaḍa dialect), Toda, and Kota – have the character of 'mixed' languages in the sense that they share various phonological, grammatical, and lexical features with Tamil, Malayalam, and Kannaḍa in an almost 'non-systematic' manner which points towards much borrowing and diffusion of features (in addition to specific later innovations, and a few typical and specific Nilagiri areal traits). This leads us to the assumption that such languages as Iṟula, Šōlega, Kuṟumba, or Paṇiya are not to be considered direct 'descendants', 'branched-off' dialects of Proto-Tamil, or Proto-Kannaḍa, or Proto-Malayalam, but rather

speech-forms which were evolved by the tribal communities – originally linguistically non-Dravidian – from ‘mixtures’ of various pre-Tamil, pre-Kannaḍa, pre-Malayalam dialects which were, some 2500-200 years ago, superimposed on these (Negrito-cum-Proto-Australoid) tribals by the conquering Dravidians. The result are tribal languages belonging undoubtedly to the South Dravidian sub-family, which are ‘more or less’ “Tamiloid” or “Kannaḍoid” or Malayalam-like (showing a few retentions of older stages of these large languages) and manifest a few typical innovations of their own and a few features which are typical only for the Nilagiri area. They may also have preserved a few (lexical) substratum (pre-Dravidian) forms. It will be precisely one of the main tasks of our future linguistic work to prove this hypothesis of the superimposition of various (‘mixed’) dialects of the Proto-South Dravidian upon the originally non-Dravidian tribes of the area.”

Kota (kfe)
Lewis et al. (2016):

1. numbers of speakers: “930 (2001 census). Ethnic population: 1,400.”
2. sociolinguistic situation: “Tamil [tam] used in home and major domains. Language shift taking place. Positive attitudes. Also use Badaga [bfq], Kannada [kan], Tamil [tam].”

Subbaiah (1985):
p. xvi: “The data for the analysis have been collected during my field studies during the months of December/January/May of 1968-71, at Kotagiri and kilkotagiri of the Nilgiris District.”
p. xvii: “The Kotagiri and kilkotagiri are two villages of Coonoor taluk and among these two, Kotagiri is a major panchayat town. The Kotas are living at a two mile distance from both the places. One can reach their hamlet by a walk of half an hour. The data for this present analysis are collected in these two above said areas. The informants of age group 25-55 are selected from these two areas and it is a well known fact that the Kotas are trilinguals. They speak Badaga and Tamil in addition to their mother tongue. Normally in the towns and markets, they talk Badaga and when they come down to the plain, they switch over to Tamil. Mostly the educated Kotas speak English now and then.” (Subbaiah 1985 does not say anything regarding the relationship of Kota with other Dravidian languages.)
Emeneau (1944):
p. v: “Among adult Kotas there is at present only one man who speaks English; no woman knows anything of it, but in the generation now children are probably a dozen boys and one girl who will attend school long enough to learn a smattering of not very useful English, and two or three of them may go higher and acquire a respectable command of the language. My chief informant for the Kota language was the solitary speaker of English, and excellent informant in many ways; his English, however, though fluent, was amazingly and frequently comically incorrect and very limited in scope.”
p. 1: “The Kotas are one of the four communities of long-standing residence in the Nilgiri Hills of South India. For a long time, until the invasion of the isolated plateau by the English and their native followers in the years following 1813, these tribes formed a local but not too aberrant version of the Hindi caste system. The Todas, at the top, are non-meat-eating pastoralists whose whole life, economic and religious, is centered on their herds of buffaloes. The Badagas, below them in the scale, are agriculturalists who produce the millet for their own and the Todas’ consumption. The Kotas, far down in the scale, are the artisans and musicians of the area, producing for

the other communities ironwork, pottery, and ceremonial music, and also practising agriculture for their own maintenance; they are eaters of eat, including beef, and even of carrion. The fourth community, the Kurumbas, are a jungle tribe, feared for their sorcery and bought off by the other communities. They live in the jungles on the precipitous slopes of the Nilgiris. The other three communities live on the plateau (which is about 40 miles long and 15 miles wide), not in three separate areas, but in a superimposed fashion, with settlements of the three communities scattered among one another haphazardly. The Kotas have seven villages and number in all fewer than 1,500 (by the census of 1931, 1,121).” (Emeneau 1944 does not say anything regarding the relationship of Kota with other Dravidian languages.)

Badaga (bfq)

Lewis et al. (2016):

1. numbers of speakers: “135,000 (2001 census).”
2. sociolinguistic situation: “Used as L2 by Irula [iru], Kota [kfe].”

Own knowledge: Also speak Kannada

Balakrishnan (1999):

p. 42: “Badagas speak a distinct language of their own, which was not discovered for a long time. Thurston (1909: 1viiii) has stated that the language of the Badaga community is Badaga which is said to be an ancient form of Canarese. However this can be considered as hotchpotch of many languages like Kannada, Kodagu, Toda, Kota and to some extent Tamil.”

p. 43: “Originally Badaga’s speech was a variety of Kannada and how it has largely varied from its original as to be classed as a separate dialect? From the above report it is clear that the Badaga community was the principal speech community in the Nilgiris hills from the 13th century A.D. As Emeneau (1967: 348) has pointed out, it might be intruded as a dialect of Kannada and it presented fascinating glimpses of diffusional relationship with Toda and Kota.”

p. 44: “Badaga language is said to be related to or a dialect of Kannada as they have some common innovations like PDr. *p- > h-, *v- > b- etc. But this hypothesis is nullified as Badaga language has prevailed many innovations shared with other SDr. languages and restrained a few specific features pertaining to it in the point of view of phonological and morphological structures.”

p. 50: “By looking at the above data the distinction between the exclusive and inclusive of the first person plural in the nominative forms, one can conclude that Badaga is very close to Kodagu language (from the structural point of view) whereas it is grammatically related to Toda, Kota and Tamil. And also it is found that Badaga is close to Toda and Kota by preserving separate pronominal suffixes for first person plural in the finite verb construction.”

p. 53-54: “It may be noticed from the above discussions that Badaga has to be grouped not only with Kannada but also with Kodagu, Kota and Toda. As it has been indicated earlier, Badaga has evolved as a separate language, its origin coming by the mixing of different related languages, and also due to the fusion of those languages into one single ethnic entity due to various socio-political reasons. It can also be noted that all other neighbouring communities speak different languages such as Toda, Kota etc. and each such language is spoken by a single caste group heredity to a common ancestral group. But the case of Badaga is quite different. It has many related sub-sects, and each sub-sect has migrated from different regions of Karnataka (including Coorg) and settled down in the Nilgiris in different periods. This might have paved the way for the development of crossbred grammatical structure in their speech and

keeping it as an independent language. Having established that Badaga is a south Dravidian language, one can observe that this is not closer to any one of the Sdr languages. Since otherwise the similarities found among these languages have to be explained as due to areal convergence in these neighbouring languages. From the foregoing observations, one can determine the position of Badaga in SDr as follows. (tree structure follows, red.)"

Pilot-Raichoor (1991):

p. 33-34: "Enfin, signalons un dernier paramètre socio-linguistique que n'est pas propre aux Badagas mais se retrouve dans de nombreuses communautés indiennes: une forte proportion de la population est bilingue (en l'occurrence badaga/tamil) beaucoup d'entre aux étant tri-lingues (badaga/tamil/anglais) ou plus. Ce phénomène rend délicate, voire impossible, la détermination du statut d'un mot comme intégré à la langue ou comme emprunt. Il apparaitra dans les textes que les locuteurs usent souvent indifféremment d'un mot ou d'une expression badaga, tamoul ou anglais pour renvoyer à une même objet ou à une même notion."

Pilot-Raichoor (1997):

p. 136: "The Badaga community is not an autochthonous tribe but has been mentioned in a report of a missionary, Father Fenicio, as inhabiting the Nilgiris since the beginning of the seventeenth century. For at least four centuries this community has lived in close connection with the other mountain tribes – the Todas, the Kotas, and the Kuṟumbas. From the beginning of the twentieth century, due to its population growth and its economic dynamism, the Badaga community has become one of the dominant social units of the Nilgiris. […] Their language, long classified as a 'dialect of Kannada', has never been thoroughly studied until now."

p. 137: "The descriptive analysis of this language revealed so many differences from the Kannada language that I was led, first, to question seriously whether Badaga is a dialect of Kannada, as it is still usually acknowledged (cf. Emeneau 1989: 137); and, secondly, to emphasize the typological similarities among the Nilgiri languages, particularly between Badaga and Ālu Kuṟumba."

p. 142: "What has now appeared fully evident is that the Badaga language and the Kuṟumba language are actually very close. (This was rightly suggested by M. B. Emeneau in 'The Languages of the Nilgiris', 1989: 138). Though they are probably derived from different backgrounds, Ālu Kuṟumba being originally more 'Tamiloid' and Badaga being more 'Kannadoid', they have both evolved in the same direction and they now look very similar in all domains: phonological, morphological and lexical."

p. 144-145: "From this quick comparison it should not be inferred that Badaga and Kuṟumba have any kind of genetic relationship. Many features show that Kurumba is more 'Tamiloid' or more archaic than Badaga; (contrast between simple and shrill ir (Kapp 1982: 23-24), a case system (Kapp 1982: 76) quite different from that of the Badaga and more Tamil-like). What these similarities express is the quick diffusion of certain features among the Nilgiri languages (most probably due to the multilingual situation), so that), independently of their genetic affiliation, they do exhibit very strong clusters of isoglosses. As far as the languages of the Nilgiri summit are concerned, irrespective of any social or ethnic affiliation, we are led to modify the picture of linguistic repartition and to propose, parallel to the Toda/Kota linguistic group, a Badaga/Ālu Kuṟumba linguistic grouping. The development of micro-areal linguistics and the careful study of the diffusion of certain Nilgiri features – those pointed out by Zvelebil (1980: 14-19) or by Emeneau (1989: 138-40) – but also many others offer an important task to pursue."

Betta Kurumba (xub)
Lewis et al. (2016):

1. numbers of speakers: “32,000 (2003 NLCI), increasing.”
2. sociolinguistic situation: “Central Institute of Indian Languages lists Betta Kurumba as endangered. It is being studied by Annamalai University. Home, work, religion. Positive attitudes.”

Zvelebil (1981):
p. 499: basically indicate that this is a “Kannadoid language’, but there are some features shared with the Tamiloid group of languages spoken in the Nilagiri hills. “The data on Bëṭṭa Kuṟumba are too meagre to allow any but extremely tentative conclusion: it seems to be fundamentally a Kannaḍoid language.” Same is repeated on page 500.
Coelho (2003):
p. 8: “The arrival of immigrant groups has pushed indigenous Nilgiri groups into social, political, and linguistic marginalization. Immigrants to the Nilgiris outnumber the population of indigenous groups. They are mainly speakers of three of India’s official languages, Tamil, Malayalam, and Kannada – state languages of Tamil Nadu, Kerala, and Karnataka, respectively, all of them part of the South Dravidian language group. Tamil has become the most dominant of these languages in the region because after Indian independence, the Nilgiris was incorporated into the state of Tamil Nadu, giving Tamil special status locally as the language that receives government support in education and employment. In addition, English plays a prominent role as the de facto national language of post-colonial India. […]The Beṭṭa Kurumbas continue to maintain their ethnic language, and children in the community all acquire it as their first language; however, they have also become proficient in Tamil, which they learn partly at school. The dominant status of Tamil and other official languages in this area poses a potential threat to the long-term viability of their own ethnic language.”
Upadhyaya (1972):
p. 307: “There are three Kuruba tribes residing in the forest ranges of the hilly district of Coorg, Mysore State. They are known as the Je:nu kuruba, A:ne kuruba, and Beṭṭa kuruba. Of these the first two speak a dialect of Kannada whereas the third, namely Beṭṭa kurubas speak a language which is not intelligible to their neighbours who speak Kannada, Kodagu or Malayalam. While surveying the Kannada dialects of this district under auspices of the Linguistic Survey Unit, Deccan Collega, Poona the attention of the present author was drawn to the existence of this interesting speech. A few samples collected from the speech of this tribe at Nagarhole, the southern-most are of this district revealed a number of features in which it differs from the neighbouring Dravidian languages and this resulted in undertaking a month’s fieldwork at Nagarhole. On analysis it was found necessary to consider it as a language belonging to the South Dravidian group, but distinct from the other well-known languages of the same stock.”
p. 326-327: “Having established this as a distinct language, our next endeavour would be to determine its comparative position and include it under one or the other sub-groups of the South Dravidian group. This language cannot be included in the Kannada sub-group as it has not changed its p to h. Its vowel structure is different from that of Kannada and has undergone many sound changes for which we cannot find parallels in Kannada. Its case suffixes and verbal suffixes are also different.”
p. 327: “The possibility of inclusion under Toda-Kota group is also ruled out. Kuruba does not have the wealth of sibilants. Nor does it drop its final vowels like Toda-Kota.

Allomorphy of the past tense suffix of Kuruba does not resemble the complicated allopmorphic system of Toda-Kota, nor does Kuruba use the past tense stem in the formation of present tense forms."

p. 327: "Absence of palatalization (of velar stops), dropping of final m from the inanimate nouns ending in am, change of v > b absence of gender distinction in third person demonstrative pronouns and the corresponding verbal forms and a number of other features noted above separate Kuruba from the Tamil-Malayalam group. Only in certain shared retentions Kuruba shows certain features common to Tamil-Malayalam."

p. 327-328: "The only language with which Kuruba can be grouped is the neighbouring language Kodagu. Though it differs from Kodagu in its verbal structure and also in not showing transitive-intransitive distinction, it shares many innovations with Kodagu. Development of retroflex vowels, change of i > ɨ, e > ë dropping of ḷ from the plural suffix may be cited as a few examples to show this. The two languages Kodagu and Kuruba constitute a sub-group within the South Dravidian."

References

Anand, R. S. 2002. Kuvi. In J. K. Banthia, B. P. Mahapatra, C. Sivashanmugam, S. S. Bhattacharya, S. P. Datta, Kakali Mukherjee, & Ranjita Nayak (eds.), *Linguistic Survey of India, Special Studies: Orissa*, 553-574.

Balakrishnan, R. 1977. *A grammar of Kodagu*. Annamalainagar: Annamalai University.

Balakrishnan, R. 1999. *Badaga: A Dravidian language*. Annamalainagar: Annamalai University.

Bhaskararao, P. 1998. Gadaba. In S. B. Steever (ed.), *The Dravidian languages*, 328-355. London: Routledge.

Bhaskararao, P. 1980. *Konekor Gadaba: A Dravidian language.* Poona: Deccan College. Publication of PhD U Poona, 1973.

Bhattacharya, S. (1976). *The tribal languages of South Kerala*. Karyavattom, Trivandrum, India: University of Kerala.

Bhat, D. N. S. 1998. Tulu. In S. B. Steever (ed.), *The Dravidian languages*, 158-177. London: Routledge.

Beine, D. K. 1994. *A sociolinguistic survey of the Gondi-speaking communities of Central India.* MA thesis, San Diego State University, San Diego.

Bouckaert, R. R. & J. Heled. 2014. DensiTree 2: Seeing Trees Through the Forest. *bioRxiv*, http://dx.doi.org/10.1101/012401

Burrow, T. & M. B. Emeneau. 1984. *A Dravidian Etymological Dictionary* [DEDR]. Second edition. Oxford: Clarendon Press. Also found online: http://dsal.uchicago.edu/dictionaries/burrow/

Chatterji, S. K. 1926. *The Origin and Development of the Bengali Language*, 3 vols. Calcutta: Calcutta University Press (Reprinted (1970), London: George Allen and Unwin Ltd).

Coelho, G. M. 2003. *A grammar of Batta Kurumba.* Ph.D thesis, University of Texas at Austin, Austin, TX.

Das, A. S. K. 1973. *Structure of Malto*. Annamalainagar, Tamilnadu, India: Annamalai University.

Ebert, K. 1996. *Koḍava*. München: LINCOM Europa.

Elfenbein, J. 1998. Brahui. In S. B. Steever (ed.), *The Dravidian languages*, 388-414. London: Routledge.

Emeneau, M. B. 1944. *Kota texts. Part One*. Berkeley: University of California Press.

Emeneau, M. B. 1955. *Kolami, a Dravidian language*. Berkeley: University of California Press.

Emeneau, M. B. 1957. Toda, a Dravidian language. *Transactions of the Philological Society* 56(1): 15-66.

Emeneau, M. B. 1984. *Toda grammar and texts*. Philadelphia: American Philosophical Society.

Fuller, D. Q. 2007. Non-human genetics, agricultural origins and historical linguistics in South Asia. In M. D. Petraglia & B.Allchin (eds.), *The evolution and history of human populations in South Asia*, 393-443. Dordrecht: Springer.

Huson, D. H. & D. Bryant 2006. Application of Phylogenetic Networks in Evolutionary Studies. *Molecular Biology and Evolution* 23(2): 254-267.

Lewis, M. P., G. F. Simons & C. D. Fennig. 2016. Ethnologue: Languages of the World, Nineteenth edition. *Online version: www.ethnologue.com.*

Mallikarjun, B. 1993. *A descriptive analysis of Yerava*. Manasagangotri, Mysore: Central institute of Indian languages.

Mishra, A. K. 1991. *Word formation in Kurukh (Oraon): a study of linguistic typology and language change.* (MA), Jawaharlal Nehru University, New Delhi.

Pilot-Raichoor, C. 1991. *Le badaga: langue dravidienne (Inde); description et analyse.* PhD thesis, Université de la Sorbonne Nouvelle, Paris.

Pilot-Raichoor, C. 1997. Badaga and its relations with neighbouring languages. In P. Hockings (ed.), *Blue mountains revisited: cultural studies on the Nilgiri Hills*, 136-147. Delhi: Oxford University Press.

Rao, B. R. 1982. Inter-relation of Tulu with other Dravidian languages. *International Journal of Dravidian Linguistics* 11: 244-252.

Southworth, F. C. 2005. *Linguistic archaeology of South Asia*. London: Routledge.

Subbaiah, G. 1985. *Grammar of Kota*. Annamalainagar: Annamalai University.

Subrahmanyam, P.S. 1998. Kolami. In S. B. Steever (ed.), *The Dravidian languages*, 301-327. London: Routledge.

Steever, S. B. 1998. Goṇḍi. In S. B. Steever (ed.), *The Dravidian languages*, 270-297. London: Routledge.

Steever, S. B. 1998. Malto. In S. B. Steever (ed.), *The Dravidian languages*, 359-387. London: Routledge.

Subrahmanyam, P.S. 1968. The position of Tuḷu in Dravidian. *Indian Linguistics* 29: 47-66.

Tyler, S. B. 1969. *Koya: An outline grammar*. Berkeley: University of California Press.

Upadhyaya, U. P. 1972. Kuruba - A Dravidian Language. In S. Agesthialingom & S. V. Shanmugam (eds.), *Third Seminar on Dravidian Linguistics*, 307-328. Annamalainagar: Annamalai Unibversity.

Zvelebil, K. V. 1981. Problems of Identification and Classification of Some Nilagiri Tribes: Iṛulas-Ūrāḷis, Kāṭṭu Nāyakas/Jēnu Kuṛumbas, Šōlegas. *Anthropos, 76*(467-528).
